# Eurados review of retrospective dosimetry techniques for internal exposures to ionising radiation and their applications

**DOI:** 10.1007/s00411-020-00845-y

**Published:** 2020-05-05

**Authors:** A. Giussani, M. A. Lopez, H. Romm, A. Testa, E. A. Ainsbury, M. Degteva, S. Della Monaca, G. Etherington, P. Fattibene, I. Güclu, A. Jaworska, D. C. Lloyd, I. Malátová, S. McComish, D. Melo, J. Osko, A. Rojo, S. Roch-Lefevre, L. Roy, E. Shishkina, N. Sotnik, S. Y. Tolmachev, A. Wieser, C. Woda, M. Youngman

**Affiliations:** 1 BfS-Bundesamt für Strahlenschutz, Ingolstädter Landstr. 1, 85764 Oberschleißheim, Germany; 2 CIEMAT - Centro de Investigaciones Energéticas, Medioambientales y Tecnológicas, Av.da Complutense 40, 28040 Madrid, Spain; 3 ENEA Casaccia Research Center, Via Anguillarese 301, Santa Maria di Galeria, 00123 Rome, Italy; 4 Public Health England - Centre for Radiation, Chemical and Environmental Hazards, Chilton, Didcot, OX11 0RQ Oxon UK; 5 Urals Research Center for Radiation Medicine (URCRM), Vorovskt str. 68A, Chelyabinsk, 454141 Russia; 6 Istituto Superiore di Sanità, Viale Regina Elena 299, 00161 Rome, Italy; 7 Cekmece Nuclear Research and Training Center Radiobiology Unit Yarımburgaz, Turkish Atomic Energy Authority, Istanbul, Turkey; 8 DSA-Norwegian Radiation and Nuclear Safety Authority, Skøyen, P. O. Box 329, 0213 Oslo, Norway; 9 SURO-National Radiation Protection Institute, Bartoskova 28, 14000 Prague, Czech Republic; 10 US Transuranium and Uranium Registries, Washington State University, Richland, WA USA; 11 Melohill Technology, 1 Research Court, Rockville, MD 20850 USA; 12 National Centre for Nuclear Research, A. Soltana 7, 05400 Otwock, Poland; 13 ARN-Nuclear Regulatory Authority of Argentina, Av. del Libertador 8250, Buenos Aires, Argentina; 14 Institut de Radioprotection et de Sûreté Nucléaire, IRSN, Pôle Santé et Environnement, Direction de la Santé, Fontenay-aux-Roses, France; 15 Southern Urals Biophysics Institute (SUBI), Ozyorsk, Chelyabinsk Region 456780 Russia; 16 Institute of Radiation Medicine, Helmholtz Zentrum München, Ingolstädter Landstr. 1, 85764 Neuherberg, Germany; 17 Chelyabinsk State University (ChelSU), 129, Bratiev Kashirinih Street, Chelyabinsk, 454001 Russia

**Keywords:** Internal dosimetry, Biological dosimetry, EPR dosimetry, Biokinetics, Internal exposures

## Abstract

This work presents an overview of the applications of retrospective dosimetry techniques in case of incorporation of radionuclides. The fact that internal exposures are characterized by a spatially inhomogeneous irradiation of the body, which is potentially prolonged over large periods and variable over time, is particularly problematic for biological and electron paramagnetic resonance (EPR) dosimetry methods when compared with external exposures. The paper gives initially specific information about internal dosimetry methods, the most common cytogenetic techniques used in biological dosimetry and EPR dosimetry applied to tooth enamel. Based on real-case scenarios, dose estimates obtained from bioassay data as well as with biological and/or EPR dosimetry are compared and critically discussed. In most of the scenarios presented, concomitant external exposures were responsible for the greater portion of the received dose. As no assay is available which can discriminate between radiation of different types and different LETs on the basis of the type of damage induced, it is not possible to infer from these studies specific conclusions valid for incorporated radionuclides alone. The biological dosimetry assays and EPR techniques proved to be most applicable in cases when the radionuclides are almost homogeneously distributed in the body. No compelling evidence was obtained in other cases of extremely inhomogeneous distribution. Retrospective dosimetry needs to be optimized and further developed in order to be able to deal with real exposure cases, where a mixture of both external and internal exposures will be encountered most of the times.

## Introduction

The European Radiation Dosimetry Group (EURADOS) is a network of more than 70 European institutions and 560 scientists dedicated to the promotion of research and development and European cooperation in the field of dosimetry of ionizing radiation (Rühm et al. 2018, 2020). The core scientific and technical activities of EURADOS are performed by a number of Working Groups covering several complementary aspects of radiation dosimetry. In 2011 the Working Group on 'Retrospective Dosimetry' (WG10) has published a review of the applications of retrospective dosimetry techniques in case of external exposures to ionising radiation (Ainsbury et al. 2011). In that context, retrospective dosimetry was defined as 'the estimation of a radiation dose received by an individual recently (within the last few weeks), historically (in the past) or chronically (over many years). The main aim of that review was to compare physical and biological retrospective dosimetry techniques for individual external exposures. Biological and EPR retrospective dosimetry methods are usually implemented in cases of external exposures when conventional dose estimation systems, e.g. personal dosimeters, are not available or reliable, and also to provide individual reassurance that overexposure was unlikely to have occurred.

Following that publication, a joint effort of EURADOS WG10 and WG7 (Working group on 'Internal dosimetry') was started in order to perform a similar overview in the case of incorporation of radionuclides and evaluate the usefulness and limitations of biological and EPR dosimetry in cases of internal and mixed internal/external exposures.

Few investigations have been carried out so far to link internal dosimetry from incorporated radionuclides with biological dosimetry methods. Biological dosimetry is well established and validated for providing dose estimations following external radiation exposures (IAEA 2011; Kulka et al. 2015; Kulka and Wojcik 2017,1). In contrast, interpreting biological dosimetry data in cases of internal exposures is challenging.

Internal exposures are generally more complex to manage than external exposures. Internal emitters indeed present peculiar aspects which distinguish them from the case of external exposures. First of all, the doses to individuals arising from internal exposures cannot be determined directly. They are inferred from measured quantities, such as body activity content, excretion rates or airborne concentration of radioactive material. The interpretation of these monitoring data in terms of intake and the lifetime Committed effective dose (delivered over 50 years)—E(50) in mSv, requires knowledge or making assumptions about a number of parameters. These are: time, type of exposure (acute vs chronic), route of the intake (inhalation, ingestion, injection, intact skin or through a wound), the physical, biological and chemical characteristics of incorporated radionuclides (e.g. type of molecular compound, particle size, solubility) and their biokinetics inside the body (Etherington et al. 2018).

An important consideration is that incorporated radionuclides are usually deposited inhomogeneously in the body and they continue to act as radiation sources until their physical decay or elimination from the body. The local dose rates thus follow complex patterns which depend on the physical properties of the incorporated radionuclide(s) (radioactive half-life, type and energy of the emitted radiation), on their chemical and metabolic properties (distribution between organs and biological retention half-lives), and on the anatomical characteristics of the contaminated subject.

The fact that the irradiation of the body is spatially inhomogeneous, potentially prolonged over large periods and variable over time makes the cases of internal exposures particularly problematic for biological and EPR dosimetry methods when compared with external exposures. Furthermore, the standard calibration curves, usually generated in vitro using only external radiation, do not apply to incorporated radionuclides, and it is still controversial against which dose (blood dose, marrow dose or total body dose) the results of the biological dosimetry assays should be tested (Ainsbury et al. 2014). The dose assessment is moreover complicated by the fact that internal exposures from incorporated radionuclides are often accompanied by external exposures from various possible sources. Each of these factors serves to increase the uncertainty on internal dose measurements, with the extent of this increase dependent on the type of exposure and dosimetry method.

The joint collaboration between EURADOS WG10 and WG7 scientists was aimed to address the above-mentioned issues and this publication summarizes the main outcomes of this study, delineating the driving lines for possible further developments in the future.

First, the manuscript briefly presents and discusses internal dosimetry methods and the most common cytogenetic techniques used for the assessment of the radiation dose in biological dosimetry. The paper gives specific information about advantages and disadvantages of each of the techniques and addresses associated uncertainties. Together with cytogenetic techniques, electron paramagnetic resonance (EPR) dosimetry applied to tooth enamel is also considered in this paper.

Based on the evidence collected in different exposure scenarios, the complementarity of the different methods and the practical feasibility and suitability of their utilization are discussed.

For this purpose, some real-life case scenarios involving internal exposures have been identified from the literature for which biological and/or EPR dosimetry has been employed and sufficient bioassay data (e.g. excreted activity rate in urine samples) are available for a reliable internal dose assessment.

For each case, the available English language literature was reviewed and summarized, the appropriateness of the employed techniques critically evaluated, the consistency between internal doses obtained from bioassay data and biological and EPR dose estimates were tested and the reasons for any encountered inconsistencies in the measurement outcomes discussed.

## Assessment of doses due to internal exposures

No physical technique is available to directly measure the dose due to internal emitters. Dose must instead be evaluated either from the knowledge of the incorporated activity or from the results of monitoring programmes assessing the activity retained (Bq) in or excreted (Bq day^−1^) from the body. This evaluation is performed on the basis of a mathematical approach which describes the metabolic behaviour of the contaminants inside the body and the delivery of dose to the organs and tissues, and requires that the initial conditions are known: route of intake, physicochemical characteristics of the radionuclides incorporated (e.g. solubility, particle size—for inhaled material; absorption from the alimentary tract—for ingested material) and the modality of incorporation (acute, chronic) (Castellani et al. 2013; Giussani 2015; Breustedt et al. 2018; Etherington et al. 2018; ICRP 2015b).

However, such information is rarely known, especially in cases of accidental intake. In these circumstances, the initial conditions can be reconstructed from the measurement of the amount of the radioactivity present in the body or excreted. Comparing the results of the measurements with the predictions of the corresponding biokinetic model, it is possible to evaluate the activity incorporated by the subject, and, under certain circumstances, also its physicochemical characteristics and the modality of incorporation. Alternatively, the incorporation event is retrospectively evaluated based on measurements or estimates of the activity concentration in the workplace and/or in the environment, and on specific assumptions on the behaviour of the contaminated subject(s). It is evident that, in particular, the second approach is affected by large uncertainties.

### Monitoring techniques

Monitoring techniques can be divided into direct methods, based on the measurement of the activity present inside the body at the moment of the monitoring (in vivo bioassay), and indirect methods, mainly based on the determination of activity removed from the body (in vitro bioassay).

Direct methods consist of the measurement, with external detectors, of the radiation emitted by the radionuclides deposited inside the body. They can only be used for radionuclides emitting penetrating radiation, i.e. X-rays, gamma radiation, positrons (by measuring their annihilation radiation) or energetic beta particles (by measuring their bremsstrahlung radiation). Besides counting geometries that enable estimation of the activity retained in the body (whole-body monitoring), partial monitoring techniques have been developed to detect the activity in thyroid (for incorporation of radionuclides of iodine), lungs (after inhalation of insoluble compounds), skull and knee (for bone seeking radionuclides) or liver (for actinides).

In vitro monitoring techniques consist of the determination of activity concentrations in the excreta or in other biological materials removed from the body. Excreta monitoring programmes usually involve analysis of urine, due to the easier implementation of this technique. Faecal analysis is recommended for those radionuclides and compounds that are primarily excreted through the alimentary tract according to their physicochemical form and the resulting biokinetics. Nose blow or nasal swab analysis can be used after a suspected incident to identify radionuclide intakes through inhalation. In special circumstances, other biological material like blood, hair and teeth can be measured. In vitro bioassay is the measurement technique of choice to quantify internal contamination of pure alpha and beta emitters, as their radiation cannot be detected from outside the body. Alpha and gamma spectrometry, mass spectrometry (i.e. ICP-MS) and liquid scintillation counting (for beta emitters) are the most common techniques employed. In many cases, pressure or microwave digestion, (radio)chemical separation and other decomposition techniques need to be applied to the samples to facilitate their measurements. The same methods are also used for autopsy samples.

### Dose assessment

#### Mathematical formulation

Assuming that the incorporated activity is known or has been retrospectively assessed, the general scheme for internal dose assessment postulates the use of biokinetic models to describe uptake, absorption, distribution, and retention of radionuclides in selected organs and tissues of the body, known as source regions. Mathematically speaking, the absorbed dose rate $\dot{D}\left( {r_{T} ,t} \right)$ to a target region *r*_*T*_ due to irradiation from all source regions *r*_*S*_ is given by the following formula:

1 $$\dot{D}\left( {r_{T} ,t} \right) = \sum\limits_{{r_{S} }} {A\left( {r_{S} ,t} \right)S(r_{T} \, \leftarrow \,r_{S} ,t),}$$

where *A*(*r*_*S*_*,t*) is the activity present in the source region *r*_*S*_ at time *t*, and is calculated with biokinetic models, *S*(*r*_*T*_* ← r*_*S*_*,t*) is the radionuclide-specific quantity representing the mean absorbed dose rate to target tissue *r*_*T*_ per unit activity present in the source tissue r_S_ and is calculated using dosimetric models and computational phantoms. In the case of incorporation of a mixture of radionuclides, Eq. (1) must be properly adjusted summing the contributions of each radionuclide.

#### Biokinetic models

Biokinetic models are mathematical models describing the distribution, retention and elimination of the incorporated radioactivity, and enable calculation of retention curves and number of transformations in each source region, as well as the profiles of activity excretion in urine and faeces. The biokinetic models describing the metabolic behaviour of radioactive contaminants inside the human body are provided and updated by the International Commission on Radiological Protection ICRP (ICRP 1993, 1994, 1995, 2006, 2015b).

The general approach of ICRP is to use compartmental structures, where specific organs and tissues are represented by interconnected compartments. The transfer and exchange of material (activity) between compartments is generally considered to be a kinetic process of the first order: the mass (activity) going from compartment *i* to compartment *j* is proportional to the mass (activity) present in *i*. For a given radionuclide, the activity curves *A*(*r*_*S*_*,t*) in the source regions *r*_*S*_ depend on the biokinetics of the radionuclide as well as on its radioactive half-life.

The models recommended by ICRP are intended to be used for radiation protection regulatory purposes, and therefore are defined as generic models for reference individuals. In case of accidental exposures, models should be properly adjusted to the individual characteristics of the exposed subjects, whenever feasible, to obtain a realistic evaluation of the dose (Castellani et al. 2013).

#### Computational phantoms and radiation transport

The radionuclide specific quantity *S*(*r*_*T*_* ← r*_*S*_*,t*) depends mainly on the anatomy of the exposed subject and on the physical characteristics associated with the incorporated radionuclide(s) according to the formula:

2 $$S(r_{T} \, \leftarrow \,r_{S} ,t) = \frac{1}{{M\left( {r_{T} ,t} \right)}}\sum\limits_{i} {Y_{i} \cdot E_{i} \cdot \phi (r_{T} \, \leftarrow \,r_{S} , \, E_{i} , \, t).}$$

Here *E*_*i*_ is the energy of the *i*th nuclear transition, *Y*_*i*_ is the yield (probability) of that nuclear transition, *ϕ(r*_*T*_* ← r*_*S*_*, E*_*i*_*, t)* is the fraction of radiation energy *E*_*i*_ emitted within the source tissue *r*_*S*_ at time *t* that is absorbed in the target tissue *r*_*T*_, and *M(r*_*T*_*,t)* is the age-dependent mass of the target tissue *r*_*T*_ in the reference individual. For intakes by workers and adult members of the public, the terms in Eq. (2) are considered to be time independent.

The term *ϕ(r*_*T*_* ← r*_*S*_*, E*_*i*_*, t)* is evaluated using Monte Carlo radiation transport codes, assuming a uniform activity distribution in the source region. For non-penetrating radiation, such as alpha particles, it is generally assumed that the whole energy is deposited in the source region. Earlier this assumption was used also for beta emitters, and the average energy of the beta emission was used. Now full Monte Carlo radiation transport calculations for beta emitters are performed using the full beta energy spectrum instead of the mean energy (ICRP 2016). Computerized representations of the organs in the body (phantoms) are required to perform these calculations for gamma radiation and energetic beta radiation. The stylized geometrical phantoms developed by Cristy and Eckerman (1987) have now been replaced by high-definition phantoms (computational reference phantoms) obtained from the segmentation of medical diagnostic images of actual patients (ICRP 2009). In specific cases, such as hollow organs or skeletal tissues additional assumptions are used (ICRP 2016).

#### Sources of uncertainty

A number of authors have considered uncertainties in assessment of internal doses. Together with many others, Etherington (2007), considers that for both in vivo and in vitro dosimetry methods, Monte Carlo modelling is a highly informative and appropriate method to incorporate information on uncertainties in intake patterns, measurements and biokinetic model parameters to create probability distribution functions for assessed intake and dose.

A publication of the National Council on Radiation Protection and Measurements (NCRP 2009) attempted to summarize knowledge about uncertainties in all kinds of internal dose calculations, for radiation workers, and nuclear medicine patients. The publication considers the range of sources of uncertainties including in personal information, bioassay and environmental measurements, intakes and the biokinetic, intake, retention and systemic, and dosimetric models. Practical examples are given for use of classical, Monte Carlo and Bayesian techniques to formally calculate uncertainty in a range of potential scenarios. Measurement methods are also detailed in appendices.

Puncher and Harrison (2012) discuss a methodology for performing the uncertainty analysis for inhaled and ingested radionuclides and review studies that quantify uncertainty on dose and risk.

A further review (Paquet et al. 2016) considers uncertainty and variability in assessment and interpretation of internal doses in detail, considering uncertainty in effective dose from the averaged nature of the tissue weighting factors, and explaining the rational for use of effective dose as dose to a Reference Person rather than for a specific individual. In recent years, however, the increased sophistication of the models means that it is now possible in some circumstances to adequately consider uncertainties in parameters and inter-individual variability. Sources of uncertainty are discussed as above, but the authors consider in particular time and route of intake—with this potentially being the dominant source of uncertainty in situations when the intake is not recognized for some time and/or retention/excretion rates quickly decrease, whereas when intake is immediately recognized and/or retention/excretion is relatively stable, time and route of intake will be a relatively minor source of overall uncertainty in estimated dose.

A practical approach for dealing with type A and type B uncertainties associated to monitoring data in case of individual monitoring programs for exposed workers is presented in EC Report 188 (Etherington et al. 2018) and EURADOS Report 2013-01 (Castellani et al. 2013).

Overall, although there has been a relatively large amount of work by a relatively small number of group and individuals in recent years, uncertainty assessment in the field of internal dosimetry remains an active field in its own right. Indeed, a recent publication by the EURADOS Working Group on Internal Dosimetry identified uncertainty in internal dosimetry as a priority for future research, as part of a wider programme to close the gaps in monitoring assessment and treatment following internal radiation contamination (Li et al. 2016).

## Retrospective dosimetry techniques

### Biological dosimetry methods

Several methods for biological and EPR dosimetry were developed to provide tools for dose assessment after known or suspected external exposure to ionizing radiation, when physical dose reconstruction is impossible or uncertain (Ainsbury et al. 2011). The most commonly used cytogenetic techniques are: the dicentric chromosome (DC) assay, the cytokinesis-blocked micronuclei (CBMN) assay, and the fluorescence in situ hybridization (FISH) translocation assay.

The three assays are based on the frequency of chromosomal damage in peripheral blood lymphocytes (PBL) originating from pluripotent stem cells in the bone marrow and differentiated in the thymus (T-lymphocytes). T-lymphocytes are long living circulating cells that can reach all body areas and can thus be considered as circulating dosimeters. According on their immunological function, T-lymphocytes can be divided into short-lived (half-lives of some weeks-few/months) or long-lived (half-lives around 3.5 years or more).

It is generally thought that roughly 80% of circulating lymphocytes survive for approximately 4.5 years, declining slowly with a half-life of 8–15 years (Hammarlund et al. 2003).

The fading of T-lymphocytes over time, causes a decrease in the number of detectable unstable aberrations, such as dicentrics. Therefore, the DC assay is most precise after external acute exposure and blood samples should be taken some days or a few weeks after exposure. If the assay is conducted several weeks or months after exposure, the elapsed time has to be considered, as the decrease in the biological indicators has an increasing impact on the uncertainty correlated with the estimated dose. In general, only stable translocations may persist over years or decades and accumulate during chronic exposures as they might arise from the bone marrow that has been exposed. Therefore, the dose to the bone marrow represents an important contribution to the dose measured on circulating lymphocytes many years after an accident.

The internal incorporation of radionuclides could be considered as a particular type of protracted and usually very inhomogeneous exposure of the body. The reason for this is that the sites of deposition of a radionuclide and its retention time are related to several factors such as: the route of entry into the body, the physicochemical characteristics, the quality of the radiation emitted, the metabolic pathways into which the radionuclide may be incorporated and the subject’s physiological status (IAEA 2011). Therefore, even if the induction of chromosome aberrations may be observed in PBL of subjects internally contaminated, many factors have to be considered to derive a meaningful estimate of radiation dose to the whole body or to specific organs.

The accuracy of dose estimation by cytogenetics is considered to be most reliable in the case of internal contamination with radionuclides dispersing fairly uniformly around the body. In such rare cases the aberration yield could be referred reliably to a dose–response curve generated for lymphocytes irradiated in vitro.

However, these are the exceptions, as most radionuclides are heterogeneously distributed within the body, with preferential uptake in specific organs and tissues, which is dependent on the individual element and its chemical behavior (Ainsbury et al. 2014).

#### The dicentric chromosome (DC) assay

Researchers have been applying the DC assay for dose assessment in case of external exposure for more than 50 years, and a huge amount of data is available. The dicentric chromosome, the main aberration used for biological dosimetry, is an abnormal chromosome produced by asymmetric exchange between the centromeric pieces of two broken chromosomes. The method is well described (IAEA 2011) and the procedures for dose assessment are standardized for both small radiation accidents (ISO 2014a) and large-scale radiation events (ISO 2008). In general, blood samples are taken shortly after exposure, the lymphocytes are cultivated for two days and 500–1000 metaphase spreads scored. The spontaneous background frequency in healthy unexposed populations is very low (approximately 1 dicentric in 1000 cells) allowing detection of whole-body acute doses around 0.1 Gy and above. Several studies have demonstrated a very good reproducible dose effect relationship for DCs, which is linear quadratic up to 5 Gy for low linear energy transfer (LET) radiations and linear for high LET (alpha or neutrons) radiation. The dicentric frequency increases similarly after in vivo or in vitro exposure, which allows establishing calibration curves for external exposures involving many different radiation qualities and various exposure scenarios.

Even if in vitro dose effect curves with different radionuclides could be performed, the simulation of radionuclide incorporation is a challenge because the dose rate is declining due to the biological and physical half-life of the radionuclide. Furthermore, the dosimetry for alpha and beta irradiation which is often restricted to intracellular effects (within the cell nucleus) is different from a gamma component, which is comparable to external radiation. If the radionuclide is homogenously distributed in the body (e.g. tritium or caesium), the peripheral lymphocytes may be well suited as dosimeters and corresponding calibration curve can be established but this is not possible for all radionuclides. Most of the radionuclides are very organ specific (e.g. ^131^I, ^224^Ra, ^90^Sr, ^239^Pu) and the detectable dose in lymphocytes is far away from the dose in the organ or target tissue of concern and this makes a dose correlation difficult or impossible.

After homogenous external exposure with low LET, the DCs follow a Poisson distribution, which allows the identification of partial body exposure. The existing models for partial body exposure are the contaminated Poisson method proposed by Dolphin (1969) and the Qdr method proposed by Sasaki and Miyata (1968). Both methods are described in detail in the IAEA EPR-Biodosimetry manual (IAEA 2011). Several new methods are in development (e.g. Higueras et al. 2016). In cases of chronic incorporation of organ-specific radionuclides, these models are of limited use.

The frequency of DCs fades with the turnover of the T-lymphocytes, as DCs are unstable aberrations preventing successful cell division. After external exposures to high doses, different half-lives are reported in the literature: e.g. 3 years (IAEA 2011; Lloyd et al. 1980), about 1.5 year (Ramalho and Nascimento 1991), only 0.5 year (Bauchinger et al. 1989) or between 4 to 10 years (Chung et al. 2000; Ainsbury et al. 2014).

**Fig. 1:**
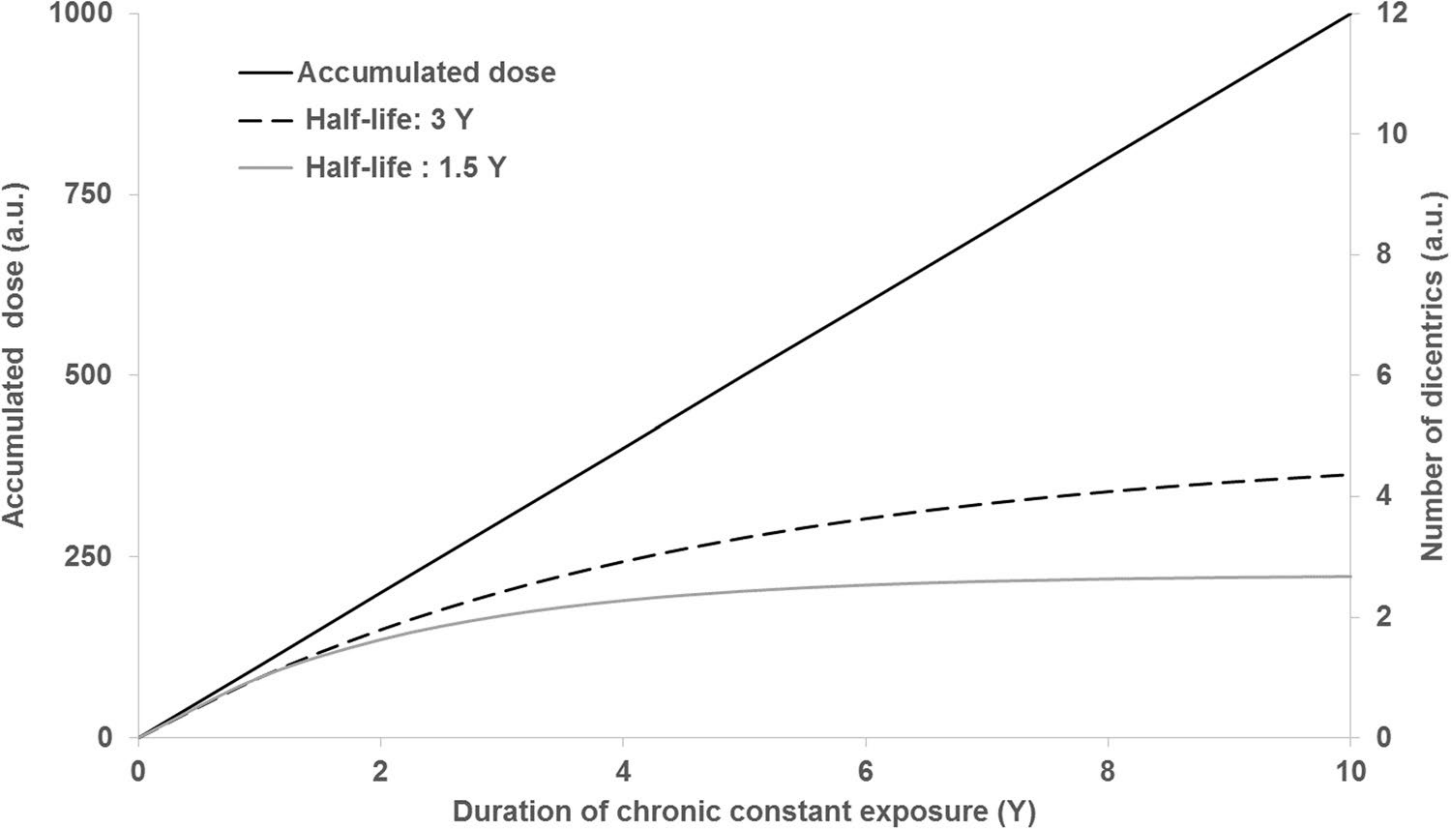
Schematic illustration of the saturation of the DC signal due to fading during chronic exposure with constant annual doses (by H. Romm). Black solid line: accumulated dose (left *y*-axis); black dashed lines: aberration yield assuming a survival half-life of the DC signal of 3 years (right *y*-axis); grey solid line: aberration yield assuming a survival half-life of the DC signal of 1.5 years (right *y*-axis)

This has consequences on the accumulation of DCs during a long-term exposure, where an increased aberration level will be detected, but mainly resulting from the exposure during the most recent years, due to the T-lymphocytes turnover. Figure 1 shows, as an example, an hypothetical case of a chronic, constant exposure, which gives to a linearly increasing accumulated dose (black solid line, left *y*-axis; values are expressed for sake of simplicity in arbitrary units). However, the resulting aberration yield (expressed as well in arbitrary units, right *y*-axis) deviates from the linear behaviour, the extent of the deviation being dependent on the value of the half-life assumed for the survival of DCs. In the figure two examples are shown assuming survival half-lives of 3 years—dashed line—and 1.5 years—grey solid line—respectively.

#### The cytokinesis-blocked micronucleus assay (CBMN)

The in vitro CBMN assay has been used for biological dosimetry since 1985 (Fenech and Morley 1985). The reliable scoring of micronuclei, which are small nuclei that form whenever a whole chromatid/chromosome or chromatid/chromosome fragments are not incorporated into one of the daughter nuclei during cell division, can be learned very easily in a few hours. Moreover, the scoring is not so time consuming as for DCs, which requires well trained scorers. Conversely, micronuclei are not as radiation specific as dicentrics, since they arise from fragments and whole chromosomes and may be induced either by clastogenic chemicals or aneugenic agents. The background frequency, therefore, presents higher individual variations and an age and gender dependency (Fenech and Bonassi 2011). Consequently, the detection limit of this assay in cases of external low LET exposure is calculated to be about 0.3 Gy whole-body absorbed dose, although the centromere staining technique could improve this detection limit as it permits screening out some of the background ‘noise’ due to non-radiological causes. As with the DC assay, the CBMN assay is well standardized (IAEA 2011; ISO 2014b) and validated as a biodosimetric tool for external acute exposure in radiation accidents up to 5 Gy. In contrast to the DC assay, the MN are inherently over dispersed which results in great uncertainties if dose assessment after partial body exposure is calculated. The micronucleus frequency fades due to lymphocyte renewal as for DCs and a half time of about one year is reported in radiation therapy patients (Fenech et al. 1990) and after accidental overexposure (Thierens and Vral 2009).

There is no reason to expect different fading after internal exposures however data are very sparse and controversial.

#### Fluorescence in situ hybridization (FISH) translocations

Chromosome translocations, i.e. the symmetrical exchange of terminal segments between two chromosomes, can be detected using fluorescence in situ hybridization (FISH), a molecular cytogenetic technique that uses fluorescent probes specifically binding to complementary parts of a given chromosome. Immediately after an acute external exposure, the frequency of radiation induced translocations in peripheral T-lymphocytes may be considered quite similar to the parallel induced frequency of DCs. As these aberrations are far more stable than the DCs, their spontaneous frequency in healthy unexposed control subjects shows a clear increase with increasing age of the donor (Whitehouse et al. 2005; Sigurdson et al. 2008). Therefore, in case of an acute, uniform whole-body external exposure, the detection limit for cumulative lifetime dose is calculated to be about 0.2 Gy whole-body absorbed dose for young subjects and 0.5 Gy whole-body absorbed dose for older people (Ainsbury et al. 2014).

The real great advantage of this assay is the transmission of stable translocations induced in irradiated bone marrow cells to circulating lymphocytes, which may result in a persisting aberration frequency over time. This characteristic feature of stable translocations opens the opportunity to identify protracted/chronic exposure or past exposures even some years or decades after the accident.

Therefore, FISH translocation is the method of choice after external long-term exposures as well as in case of chronic exposures, because it is the only method able to highlight aberrations deriving from proliferating stem cells into peripheral blood lymphocytes. The frequencies of translocations are expected to increase during chronic exposures, representing the accumulated dose in the bone marrow.

The established calibration curves, constructed from brief in vitro irradiation of blood, cannot simulate long term exposure over many years as it is described for some bone seeking radionuclides. In such kinds of protracted exposure, the dose estimation would be based on the linear term of the calibration curve, which represents the radiation quality. The quadratic term can be neglected at very low dose rates, but the resulting dose assessment is a blood dose. This may be of some interest as it can feed into biokinetic models, but can be totally different from the dose estimate that is really wanted; namely that to the target organ(s) and tissues that are particularly irradiated by the incorporated radionuclide.

As well discussed by Ainsbury and co-authors (Ainsbury et al. 2014), in cases of radiation exposures from internal emitters that irradiate specific target tissues, the question of what FISH is able to measure is not easy to answer. At any one time, the human body contains a mixed population of mature circulating CD4 and CD8 T-lymphocytes, lymphocyte stem cells and progenitors in the bone marrow which will pass to the thymus for maturation, and immature T-cells which were originally seeded in the thymus during thymopoiesis. The proportions of these populations could depend also on the developmental stage of the individual as well as the individual’s immunological history.

In the particular case of bone-seeking internal emitters specifically incorporated in close proximity to, or indeed within, the bone marrow (e.g. ^90^Sr and ^239^Pu), it is likely that the precursors of the population of sampled lymphocytes would be irradiated as precursor cells in the bone marrow and several decades after the radiation exposure, most of the lymphocytes in the blood would be descendants of these precursors.

A great complexity of factors is involved, on a case-by-case basis, in different internal contamination scenarios and this must be considered for a realistic dose assessment and evaluation of the uncertainties.

#### γ-H2AX assay

A radiation-induced double strand break (DSB) is always followed by the phosphorylation of serine 139 of the histone H2AX, a variant of the H2A protein family. This newly phosphorylated protein, γ-H2AX, represents the first step in recruiting and localizing DNA repair proteins (Sedelnikova et al. 2003). Specific fluorescent markers for this phosphorylated form (γ-H2AX), allow the visualization of foci at the sites of DSBs formation (Roch-Lefèvre et al. 2010).

The main advantage of the γ-H2AX assay is that samples can be processed and analyzed immediately, but, the rapid disappearance of the signal with time (few hours) after exposure, represents the major limitation of this assay in retrospective dose assessment.

In vitro, the γ-H2AX foci appear very quickly in lymphocytes within minutes after irradiation in a dose-dependent manner, reaching a peak in 30–60 min and then decrease to background levels within the first 24 h (Beels et al. 2010).

Apart from retrospective evaluation of the absorbed dose in cases of accidental exposure to IR for which it seems to be generally not very useful, the γ-H2AX assay has widespread clinical and preclinical applications on translational cancer research to measure the biological effects of DNA damaging agents used in both chemotherapy and radiotherapy (Ivashkevich et al. 2012). Another important potential application of γ-H2AX assay is the assessment of individual radiosensitivity of prospective patients by monitoring normal tissue responses in parallel with the clinical cancer outcome (Ivashkevich et al. 2012).

#### Uncertainties in retrospective dosimetry techniques

The current status of uncertainty assessment for general application of biological and physical retrospective dosimetry techniques has recently been reviewed (Ainsbury et al. 2018), and thus this topic will not be considered in detail here.

In brief, it was observed that the approaches used in the process of uncertainty assessment vary between different dosimetry techniques and, moreover, the overall effort devoted to uncertainty analysis varies widely between groups of retrospective dosimetry practitioners (Ainsbury et al. 2017). It was concluded by the authors that although sufficient techniques are available and in use by most laboratories for acute, external whole-body exposures to highly penetrating radiation, further work will be required to ensure that statistical analysis is always wholly sufficient for the more complex exposure scenarios including those involving internal contamination.

### EPR dosimetry

EPR dosimetry is based on the quantification of stable radicals induced by ionizing radiation in solid matrices. The EPR signal intensity is a measure of the radical concentration within the material volume and it is proportional to the volume averaged dose. Tooth enamel is the biological tissue used in scenarios with internal contamination.

The history of EPR dosimetry with calcified biological tissues started when Gordy et al. (1955) performed the first EPR detection of radiation-induced radicals in an X-ray-irradiated skull bone. A few years later, Cole and Silver (1963) described the first observation of radiation-induced EPR signals in human teeth. Because tooth enamel, after eruption, is a mineral material, the radiation- induced radicals are trapped in the matrix and so remain very stable over time (up to 10^7^ years).

Nowadays, EPR dosimetry with tooth enamel is a well consolidated method (IAEA 2002; Fattibene and Callens 2010). Several comparisons between laboratories have demonstrated that doses between 0.1 and 0.2 Gy can be reconstructed with an uncertainty of 30% or less. The detection limit has been demonstrated, in an inter-laboratory comparison, to be lower than 0.1 Gy for 50% of the participants and lower than 0.2 mGy for 70% (Wieser et al. 2008; Fattibene et al. 2011).

The dose obtained by EPR is conventionally dose to the material, i.e. to tooth enamel, where the composition of tooth enamel is assumed to be that of hydroxyapatite. When generated by external exposure, such dose can be converted to air kerma and then whole-body dose by dose conversion factors (Ulanovsky et al. 2005). When from internal contamination, it is dose to tooth enamel and does not refer to any organ.

EPR dosimetry allows the evaluation of the accumulated absorbed dose, generated by the lifetime environmental, medical and occupational exposure, both external and internal (Fattibene and Callens 2010). However, all types of exposure generate the same radical species in tooth enamel, so that internal and external radiation contribution can be distinguished only by making use of an independent method to measure the internal radioactivity, upon which the internal dose is calculated. The external dose contribution is then obtained by subtracting the calculated internal dose from the cumulative dose.

The method of internal dose calculation depends on radionuclide behavior in organism. For example, caesium isotopes, which behaves like potassium, are distributed uniformly within soft tissues and γ-ray emission is a primary source of enamel exposure (Borysheva et al. 2007). The in vivo radionuclide measurements can be performed with whole-body counters (WBC) (Shishkina et al. 2017). Photons of thyroid-seeking ^131^I may also contribute to enamel doses. The radionuclides that are particularly important for EPR dosimetry are the isotopes of those elements which constitute the tooth. These are the radioactive isotopes of calcium (^45^Ca), phosphorous (^32^P) and other trace elements, such as lead, radium and strontium, which can substitute for calcium (Driessens and Verbeeck 1990).

Methods that have been frequently used to measure independently the radioactivity in the tooth are the thermoluminescence-based beta dosimetry (Shishkina et al. 2005; Veronese et al. 2008; Shishkina 2012), the scintillating detectors (Tikunov et al. 2006), the measurement of ^90^Sr with a whole-body counter or the gas-flow Geiger-Muller detectors (Tolstykh et al. 2000, 2003). The absorbed dose to tooth enamel from the internal contamination of radionuclides in the enamel itself or in the nearby tissues, is then estimated by using computational phantoms and dosimetric models (Volchkova et al. 2009; Ferrari 2010; Shishkina et al. 2014).

In case of internal contamination, bone-seeking radionuclides may incorporate inside the tooth enamel itself or in the dentine, though with two different paths. Tooth tissues incorporates the radionuclides mainly during the process of hydroxyapatite (HP) crystalline formation. However, the time of calcification is different for enamel, primary crown dentine and secondary crown dentine. Maximum rate of HP growth in enamel and primary dentine is before the tooth eruption. Afterwards, the radionuclide concentration does not change very much: elimination is caused mainly by radioactive decay; further incorporation may be caused only by the ion transport. Secondary dentine grow during lifetime filling the pulp cavity and may accumulate activity in high concentrations (Shishkina et al. 2014).

The dose to tooth enamel from the radioactivity in enamel and dentine is relevant. Bone-seeking radioactive elements can be also incorporated in maxillary and mandible bones and act as external sources for the tooth enamel. The exposure from the radionuclides incorporated in bones contributes to a low extent to the EPR signal in enamel (Shved and Shishkina 2000). The dose to tooth enamel from the radioactivity in dentine is more relevant. Performing EPR parallel measurements in dentine and enamel may give information about the time of the contamination.

## Scenarios involving incorporation of radionuclides

Radionuclides can be incorporated into the human body by inhalation, ingestion, injection or a wound. Occupational internal exposures to radioactive materials may result from working in the nuclear industry (e.g. in fuel production and reprocessing plants), in scientific research facilities using radio-labelled compounds, during the manufacture, manipulation and clinical use of radiopharmaceuticals and from exposure to other unsealed radioactive sources. Internal exposures to the population occurred as a consequence of nuclear accidents or large-scale radiation events like those at the Chernobyl Nuclear Power Plant (IAEA 1992), at Goiânia (IAEA 1988, 1998; Oliveira et al. 1998; Rosenthal et al. 1991), in the Techa River region (Degteva et al. 2012, 2015; Shagina et al. 2012), and in the surroundings of atomic bomb tests sites (e.g. Semipalatinsk: Gusev et al. 1997). Beyond occupational and accidental exposures, medical procedures contribute significantly to the internal exposure of the population, due to the constantly increasing number of diagnostic and therapeutic uses of radiopharmaceuticals in nuclear medicine.

The following cases were found to provide relevant information for the purpose of this study:

- accidental intake of ^137^Cs occurred in Goiânia;
- occupational exposures to tritium (^3^H);
- exposures to plutonium for Manhattan Project workers, workers from the Rocky Flat Plant in Colorado (USA) and Mayak workers (from the former Soviet Union);
- exposures of patients administered with Thorotrast (^232^Th);
- exposures to thorium for workers in the NORM industry;
- exposures of patients treated with radioiodine (^131^I);
- intake of ^239^Pu, ^137^Cs and ^89,90^Sr by the populations living around the Semipalatinsk test site;
- intake of ^89,90^Sr and ^137^Cs by the population living near the Techa River.

In the English language literature, there is a lack of information regarding the Chernobyl NPP accident relevant for this publication. The report of the International Chernobyl Project (IAEA 1991) provides some information about internal caesium doses for some populations based on the WBC measurements performed by an international team in 1990. There is also information on the estimation of internal doses based on the environmental transfer through the food chain of ^90^Sr and caesium isotopes, dose assessments based on the deposition density of caesium, strontium and iodine, as well as based on the content of radioactivity in milk and leafy vegetables. However, there is no available information about attempts to assess the doses to these populations by retrospective dosimetry techniques. This is the reason why this scenario was not included in the list of case studies.

In the following sections, the selected scenarios are presented and discussed. Each scenario is characterized by the radionuclides that were incorporated and their emissions (alpha, beta and gamma), the route of intake (inhalation, ingestion, etc.), the type of exposure (acute vs. chronic, occupational vs. public), the simultaneous presence of possible sources of external exposure.

For the sake of simplicity, the case studies here presented have been divided into two groups, one representing internal exposures after incorporation of a single radionuclide and one representing exposures after incorporation of mixtures of several radionuclides.

### Intake of a single radionuclide

#### Exposure to ^137^Cs

Caesium-137 (^137^Cs) is a beta and gamma emitter with a half-life of 30.05 years. It is considered to be rapidly absorbed to the blood when inhaled (absorption type F) or ingested (ICRP 1993, 2018). The distribution of caesium in the human body does not differ greatly for different tissues (ICRP 1993, 2018; Leggett et al. 2003). Caesium-137 is usually easily detected using in vivo monitoring techniques (WBC) by gamma-ray spectrometry.

The most relevant accidental intakes of caesium occurred in Goiânia, Semipalatinsk and on the Techa River. In the latter two cases, incorporation of ^137^Cs was accompanied by other internal and external exposures and these scenarios are addressed later in this manuscript. Here we focus on the Goiânia incident.

This radiological incident occurred in 1987 at Goiânia, in the Brazilian state of Goiás (IAEA 1988, 1998; Rosenthal et al. 1991). A ^137^Cs source of 50.9 TBq used for teletherapy was stolen from the premises of an abandoned private clinic. The source was in the form of caesium chloride salt, which is highly soluble and readily dispersible. In the attempt to dismantle the head of the therapy unit, the source capsule was ruptured, leading to a widespread external irradiation from skin contamination accompanied by ingestion of ^137^Cs. The blue glow bremsstrahlumg light from the source fascinated several persons, and fragments of the source of the size of rice grains were distributed to several families. It was only about one week later that the symptoms shown by the exposed people were associated with ionizing radiation and adequate countermeasures were set in place. In total, 112,000 persons were monitored of whom 249 were found to be contaminated externally and/or internally. Twenty persons were identified with symptoms of hematopoietic acute radiation syndrome (ARS), they were hospitalized for treatment of ARS as well as for administration of Prussian Blue (PB) to enhance the elimination of ^137^Cs from the body. Four of them died within four weeks of their admission to the hospital. A total of forty-six individuals, internally contaminated with ^137^Cs, had PB therapy in dosages from 3 to 10 g·day^−1^ for adults and adolescents and from 1 to 3 g·day^−1^ for children.

More than 20 publications were reviewed for the present analysis, the majority written by staff from the Instituto de Radioproteҫão e Dosimetria, in Rio de Janeiro, Brazil. Internal doses to contaminated subjects were assessed on the basis of their ^137^Cs body content and specific cesium retention parameters, including the efficacy of PB in enhancing the cesium elimination from the body, as determined by means of whole-body measurements. The factor of reduction of committed dose due to the action of PB ranged from 1.6 to 6.1 (Melo et al. 1994; IAEA 1998; Lipsztein et al. 1998). The IAEA report gives committed internal doses for 50 individuals which ranged from 0.3 mGy to 0.4 Gy for those individuals not submitted to PB therapy; and ranged from 4.6 mGy to 0.97 Gy for those individuals treated with PB; the range would have been from 39 mGy to 5 Gy without PB therapy. Doses determined using cytogenetic dosimetry were reported in a large number of papers, for example Ramalho et al. (1988, 1991, 1996), IAEA (1998), Natarajan et al. (1998), Oliveira et al. (1998). Doses for 129 people were estimated through analysis of the frequencies of chromosomal aberrations (dicentric and centric rings), with 24 subjects exceeding 0.5 Gy and 15 exceeding 1 Gy (Lipsztein et al. 1998; IAEA 1998). Individual dose estimates were reported for 30 people in the IAEA report (IAEA 1998), and ranged from 0.1 to 5.3 Gy. The half-time of unstable chromosomal aberrations’ elimination was found to be dependent on the dose. The mean value of 110 days was found for individuals who received doses higher than 1 Gy; and the mean value of 160 days was found for individuals who received doses lower than 1 Gy. After 7.5 years, the frequencies of dicentric and centric rings were similar for individuals that had received doses between 0.1 to 2.8 Gy (Lipsztein et al. 1998).

**Fig. 2:**
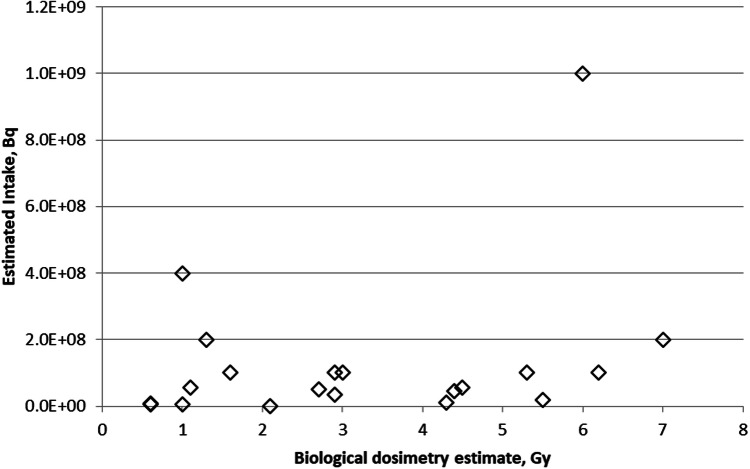
Comparison of doses estimated by cytogenetic dosimetry and assessed intake of ^137^Cs (data from Brandão-Mello et al. 1991)

Figure 2 compares whole-body absorbed doses determined using cytogenetics and estimated intakes of ^137^Cs for 20 individuals who were administered PB (Brandão-Mello et al. 1991). Some of these individuals were also treated with granulocyte–macrophage colony-stimulating factor (GM-CSF). There is no significant linear correlation between biological dosimetry estimates and intake of ^137^Cs (Pearson's *r* = 0.2925, *p* = 0.21).

There are three references (Melo et al. 1994; Ramalho et al. 1988; Natarajan et al. 1998) which directly compare estimates obtained by cytogenetic dosimetry and internal doses from the intake of ^137^Cs. Ramalho et al. (1988) present data for 29 subjects who received at least 0.5 Gy whole-body absorbed doses according to the cytogenetic dosimetry. These values are compared with the committed dose delivered until blood collection for cytogenetic analysis. Essentially, the same subjects are presented in Natarajan et al. (1998); however, in this second paper biological dosimetry was performed by assessing translocation frequencies by means of FISH, and the doses are consistently approximately 25% lower than those presented in Ramalho et al. (1988). Biological and committed internal doses for the same individuals are also reported in different tables in (IAEA 1998) and also in a separate publication (Lipsztein et al. 1998).

**Fig. 3:**
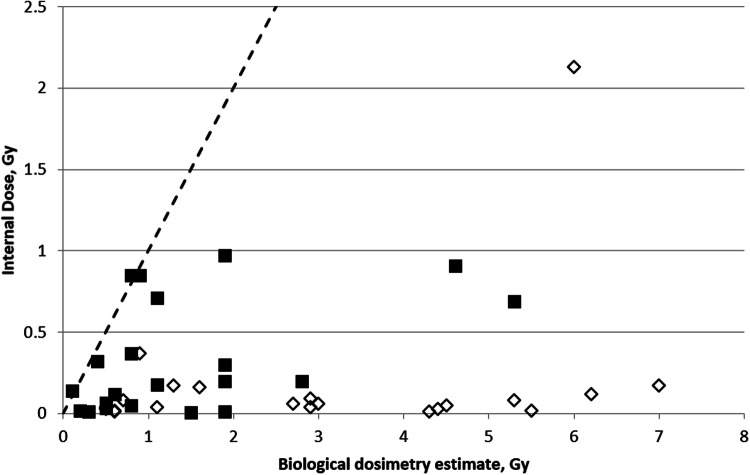
Comparison of doses estimated by cytogenetic dosimetry and committed internal absorbed dose estimated by whole-body measurements. White diamonds: data from Ramalho et al. (1988); Black squares: compilation of data from Melo et al. (1994), Tables 7.1, 7.2 and 8.2 of IAEA (1998) and Lipsztein et al. (1998). The dashed line represents the identity relation

A compilation of these data is presented in Fig. 3. For the sake of comparison also a dashed line, representing the identity expression, is given. The comparison shows that there is no consistency between internal and biological dosimetry estimates. Only 4 individuals show a reasonable agreement between the two estimates (data close to the identity line): for the remainder of the subjects biological dosimetry provides values which are a factor of between 2 and 430 times higher than the corresponding committed internal doses, although these have been calculated including also the contribution received after blood collection.

A likely explanation of the deviations observed is that the cytogenetic estimates are largely influenced by doses from external exposure, which were greater than those from internal exposure in most cases for this accident. A number of confounding factors should also be considered: some victims were treated with GM-CSF, had received transfusions and/or were treated with Prussian blue, thus reducing the internal dose. The impact of these treatments on the biological dosimetry estimate is not known.

#### Exposure to tritium (^3^H)

Tritium (^3^H) emits beta particles with 19 keV maximum energy, its radioactive half-life is equal to 12.3 years. Tritium is easily detected by liquid scintillation counting (LSC) and intakes are evaluated from the excretion rate in urine samples. Tritium mainly exists in the environment as tritiated water (HTO) or in organically bound form (OBT). Depending on its chemical form, tritium is expected to have a different biological effect and, therefore, influence the retrospective dose evaluation.

The calculation of effective dose is possible for HTO but depends on the binding compound for OBT. For HTO several assumptions are made. Following intake of tritium, HTO is instantaneously translocated to blood to mix rapidly with total body water, leading to relatively fast, uniform distribution around the body. The calculation of the effective dose from urinary concentration of tritium is based on the standard ICRP models and is described in Lloyd et al. (1986).

In vitro calibration curves are established mixing tritium with lymphocytes. Seven publications (Broome et al. 2002; Deng et al. 1998; Hori and Nakai. 1978; Prosser et al. 1983; Ribas et al. 1994; Tanaka et al. 1994; Vulpis 1984) have established calibration curves for cytogenetic damage (unstable aberrations, translocations, micronuclei) following in vitro tritium exposure with tritiated water. The shape of the curve is not commonly accepted but experimental conditions vary from one study to another. Furthermore, adjusting the concentration to have a range of doses has an impact on the dose rate, which is not considered in most studies. To obtain average doses to the whole body, the doses to lymphocytes are therefore be multiplied by a factor, which takes into account the ratio of water in the lymphocytes to the water of the body.

Lloyd et al. (1986, 1998) report one case of accidental overexposure to tritiated water providing interesting input on biological dosimetry applied to internal contamination.

One person (worker A, female) incorporated approximately 35 GBq and was treated by forced diuresis. Urine samples from 4 to 50 days after the incident were taken for in vitro monitoring by LSC; a saturation effect in the excretion was observed after 40–50 days. Dose assessment was carried out using urine analysis resulting in a committed effective dose of 0.47 Sv (± 20%). The dose equivalent to soft tissue is assumed equal to the effective dose equivalent.

Dose assessment of worker A was also performed by means of cytogenetic dosimetry (DC assay). Repeated blood samples were collected between day 40 and 50 after incorporation. The yield of dicentrics observed (*Y*) is converted to the dose to the lymphocytes using a previously established in vitro calibration curve (*Y* = 5.37 × 10^−2^ D). The maximal dose measured 1-month post-exposure is 0.63 Gy (Table 1). It can be noted that 178 days post-exposure dicentrics might have slightly declined whereas the cumulative dose is still increasing. Owing to this dicentric decrease with time post-exposure, the authors propose to average the doses measured at day 39 and day 50 post-exposure, leading to a dicentric dose of 0.58 ± 0.10 Gy. A factor of 0.66 was used to convert this absorbed dose into the effective dose equivalent, taking into account the percentage of water in the lymphocytes (0.82%) and in the body (0.54% for females). The result of 0.39 Sv (95% confidence limit: 0.28–0.48) is slightly lower but very close to the dose estimated from urine measurement. Other evaluations have been performed for worker A, six and eleven years after the exposure measuring translocations by fluorescence in situ hybridization (FISH) (Lloyd et al. 1998). The corresponding doses to the lymphocytes (0.74 ± 0.14 Gy and 0.76 ± 0.12 Gy, respectively) are not statistically different from the dose derived from dicentric observations. This result is consistent with another evaluation done by Deng et al. (1998) using a new linear calibration curve established for translocations: 0.68 ± 0.06 Gy. It might be possible that the higher dose measured with translocation when compared with dicentrics, reflects the stable translocations produced by tritium in the bone marrow cells and measured several years later in the derived lymphocytes.

**Table 1:** Estimated doses received at various times after accidental intake of tritium (from Lloyd et al. 1986, 1998)

	Sampling time (days after accident)	Absorbed dose from blood (Gy)	Effective dose equivalent from blood (Sv)^b^	Committed dose to soft tissue from urine (Sv)
Subject A	4	0.41	0.27	0.21
	18	0.54	0.35	0.37
	39	0.63	0.42	0.44
	50	0.56	0.37	0.46
	178^a^	0.56	0.37	0.47 (± 20%)
	6 years	0.74 ± 0.14		
	11 years	0.76 ± 0.12		
Subject B	4	0.19	0.15	0.006
	18	0.19	0.15	0.011
	50	0.17	0.14	0.013
	178	0.17	0.14	0.014

^a^As shown by the urine data, virtually all the committed dose had been delivered by day 50. Due to the possible decline of lymphocytes with time, 178 days post-exposure is not the optimal time point to compare the doses obtained by urine analysis and the measurement of dicentrics

^b^Calculated from the absorbed dose to lymphocytes (second column) as described in the text

No further evaluation of tritium in urine has been conducted to evaluate the cumulative dose 11 years post-contamination. However, it can be concluded that in this case where 35 GBq of tritium has been inhaled, the doses assessed from cytogenetic assays and urine measurements are very close although not equal.

Dose estimation from urine measurements and cytogenetic analysis in blood were also conducted for worker B, who was exposed to the same tritiated water. For this worker a tenfold difference was observed between the cytogenetic dose and the urinary analysis. Some hypotheses are raised to understand why but no real conclusion is given by the authors to explain such difference. However, with the assumptions discussed above, it is likely that uncertainties in both the cytogenetic methods and the models used were relatively large in this case.

Summarizing all data, the final assessment of the dose to soft tissues from urine data (assumed to be equal to effective dose) is 0.47 ± 0.09 Sv, whereas the whole-body dose assessed from dicentric assays + FISH is equal to 0.72 ± 0.09 Gy (Lloyd et al. 1998). The original assessment, based only on dicentrics, provided a value of 0.58 ± 0.10 Gy (Lloyd et al. 1986). Furthermore, the contribution of uncertainties in the measured yields of dicentrics and translocations is about equal to the contribution due to the uncertainties in the slopes of the respective dose response curves (Lloyd et al. 1998).

Another study reports in vivo measurements of both dicentrics and micronuclei in two groups of 12 workers occupationally exposed to organically bounded tritium, either through the use of luminous paints or in the weapons industry (Joksić and Spasojević-Tisma 1998). The concentration of tritium in urine ranged between 1.35 and 9.43 MBq L^−1^ in the first group, and tritium was not detected in urine of the second group. Cumulative dose from tritium concentration in urine has not been calculated for either groups due to the lack of information regarding the amount and the time point of intake. This calculation is also impossible to conduct as bioaccumulation data for this OBT form are not available.

A comparison was conducted between cytogenetic damage and the concentration of tritium in urine. For radioluminous dial painters, a significant increase in the yield of cytogenetic damage (dicentrics or micronuclei) was correlated to the tritium concentration in the urine (*R* = 0.82 and *R* = 0.73 for dicentrics and micronuclei respectively).

Heterogenous results are obtained depending on the case studied. In some exposure situations, a rather good correlation between cytogenetic evaluation and doses evaluated from tritium concentration in urine is found. However, this is only based on two human in vivo studies. Further studies would be needed; especially, to account for different forms of OBT.

#### Exposure to ^239^Pu

Plutonium is a typical alpha-emitting radionuclide, highly radiotoxic especially when incorporated as an oxide. In vitro bioassay of excreta samples by alpha spectrometry or mass spectrometry (^239^Pu) are the preferred monitoring methods used for the calculation of intake and committed effective dose in case of internal exposures. For plutonium absorbed to blood the main sites of deposition are the liver and the skeleton. The current biokinetic model of plutonium is presented in ICRP Publication 67 (ICRP 1993).

##### Plutonium workers

Among studies of U.S. plutonium workers and Mayak workers, an increase in the frequency of chromosome aberrations with increasing dose was generally observed. Workers with internally incorporated plutonium were generally also exposed to external radiation during their careers; however, some authors provided evidence that increased aberration frequencies were not caused by external radiation alone, rather they were attributed to the internally incorporated plutonium. Some studies demonstrated a relationship between the yield of aberrations and plutonium body burden/alpha-dose to red bone marrow (RBM), but to establish a precise dose–effect correlation further studies are needed.

##### US studies

No excess of chromosome abnormalities was found among 25 Manhattan Project workers who had 5–420 nCi (0.19–15.5 kBq) of plutonium deposited in their bodies (Hempelmann et al. 1973). However, three studies on workers from the Rocky Flats Plant in Colorado found an elevated frequency of chromosome aberrations in the lymphocytes of plutonium-exposed workers (Brandom et al. 1979, 1990; Livingston et al. 2006). The Rocky Flats Plant was a manufacturing facility that produced plutonium triggers for nuclear weapons and recovered plutonium from retired weapons (ORAUT 2007). The manufacturing work involved machining of plutonium for weapons components, and workers were potentially exposed to plutonium via contaminated wounds as well as inhalation. Plutonium fires were a common hazard at Rocky Flats. Most of them were small and contained however, significant fires occurred in 1957, 1965, and 1969 (ORAUT 2007).

Trypsin G-banding and C-banding techniques were applied to stain chromosome preparations from 345 Rocky Flats workers and 68 controls (Brandom et al. 1979). Workers were divided into subgroups based on their systemic plutonium burden, which ranged from a positive detection to > 40 nCi (1.48 kBq). Authors found a significant increase in complex and total chromosome aberrations with increasing exposures to plutonium. They were unable to evaluate the relative contribution of external dose, but comparison of the two most highly exposed groups (groups 5 and 6) suggests that the observed increase was not due to external radiation alone. Group 6 had a higher mean estimate of incorporated plutonium (119.44 nCi = 4.42 kBq) than group 5 (26.43 nCi = 0.98 kBq), and also a markedly higher yield of aberrations than group 5. However, workers from group 6 received a lower mean cumulative external radiation dose (14.9 rem = 0.15 Sv) than did group 5 (33.3 rem = 0.33 Sv). They did, however, observe an increase in the frequency of chromosome-type aberrations among workers who had systemic plutonium burdens of 740 Bq or more. The authors suggested that a significant increase was not observed in plutonium workers who had systemic burdens less than 740 Bq due to the limited sample size. The workers considered in this study were also exposed to a variety of toxic chemicals.

FISH-based methods were used to examine stable aberrations on 47 exposed Rocky Flats workers and 26 controls. Exposed workers were divided into high and low-dose exposed and the control group consisted of employees with no occupational exposures. Workers in the low-dose group were exposed only to external radiation and had a whole-body median absorbed dose of 22 mSv, assessed through the Rocky Flats health physics records as the dose equivalent at a depth of 1 cm in the tissue of the body. Workers in the high-dose group were exposed to both internal and external radiation. Internal doses were calculated for individuals known to have internal depositions of plutonium or americium based on historical and modern bioassays (urine) and in vivo (lung) measurements as part of the medical surveillance program. Assessments of internal doses were performed using the software CINDYq (code for internal dosimetry), based on the biokinetic models and dosimetry methods recommended by the ICRP, choosing the dose equivalent to the red bone marrow as the metric for this study. It has been calculated that the high-dose group workers received median doses of 168 mSv to the bone marrow and 280 mSv from external exposure (Livingston et al. 2006). A significantly higher chromosome aberrations frequency was observed in the high-dose group when compared with the control and low-dose groups for the following endpoints: two-way and total translocations, colour junctions, S-cells and S-complex cells. A linear regression determined that the frequency of total and two-way translocations correlated to bone marrow dose, but not to external dose. This observation led the authors to conclude that the effects in the high-dose group were mainly due to doses to the bone marrow from internally incorporated radionuclides. Additionally, the authors observed no significant differences in the frequency of micronuclei in binucleated lymphocytes when the high-dose, low-dose, and control groups were compared.

##### Mayak workers

Mayak production association (PA), the first Russian nuclear enterprise for production of the weapon grade plutonium, comprises reactors, radiochemical and plutonium facilities as well as auxiliary departments. Mayak workers were exposed to external, internal and mixed exposures at wide dose range due to imperfection of technological process and protective means, as well as a lack of knowledge on radiation effects on the human body during the first decade of the Mayak PA operation since 1948. In this connection the Mayak workers cohort is the unique one to assess radiation risks in terms of prolonged exposure.

The majority of cytogenetic studies in the Mayak workers cohort demonstrated that ^239^Pu has well-expressed genotoxic effects in terms of the total number of aberrations and number of dicentric chromosomes which correlated with total absorbed RBM dose from internal alpha-radiation due to incorporation of ^239^Pu (Okladnikova 1976; Okladnikova et al. 1994, 2005, 2009).

Case follow-up studies on chromosomal changes in peripheral blood lymphocytes among patients with CRS (chronic radiation syndrome) revealed an increase in the yield of stable chromosomal aberrations (pericentric inversions, translocations) in ^239^Pu “carriers” compared with their earlier samples (Okladnikova 1985).

A cytogenetic study on Mayak workers conducted 30–35 years after CRS was diagnosed, demonstrated that the number of multi-aberrant cells was greater in individuals with a ^239^Pu body burden greater than 1.48 kBq as compared to those exposed to gamma-ray only and/or those having ^239^Pu body burdens less than 1.48 kBq (Okladnikova and Burak 1993). However, these differences were not significant.

Long-term clinical and cytogenetic follow-up for a Mayak worker with acute ^239^Pu incorporation (83.6 kBq) due to intake through a contaminated wound showed that the yield of chromosomal aberrations increased with time since the accident (Okladnikova et al. 2004). Analysis of peripheral blood lymphocytes conducted 8 months after the accident did not reveal any chromosomal aberration. A study conducted 8–10 years after the accident revealed 1–4 chromosomal aberrations per 100 cells. A further study conducted 20–22 years after the accident, showed that the yield of unstable chromosomal aberrations was 12.2 per 100 cells; the yield of stable chromosomal aberrations was 16.3 per 100 cells. By the end of follow-up, the yield of chromosomal aberrations increased up to 47.6 per 100 cells, the number of cells with complex chromosomal aberrations also increased (3.5%).

Modern techniques of chromosome staining applied on peripheral blood lymphocytes of individuals exposed to densely ionizing radiation in vivo*,* revealed chromosomal aberrations specific for high LET radiation. Thus, the mBAND study on 31 Mayak workers (Hande et al. 2003) revealed that the yield of intra-chromosomal aberrations was greater in subjects exposed to internal alpha-radiation at total absorbed RBM doses more than 0.4 Gy as compared to those exposed to external gamma-rays only at high doses (total external gamma-dose to RBM more than 1.5 Gy). Another cytogenetic study on 79 Mayak workers using mBAND revealed a linear relationship between the yield of intra-chromosomal aberrations and internal alpha-dose to RBM as well as to the assessed ^239^Pu body burden (Sotnik et al. 2011).

Hande et al. (2005) showed, that the mean yield of complex chromosomal rearrangements in plutonium workers with high ^239^Pu body burdens, equal to (2.9 ± 0.4)%, was significantly higher as compared to reactor workers exposed to external gamma-rays only ((0.21 ± 0.1)%), to moderately exposed plutonium workers ((0.2 ± 0.1)%) and controls (0%). A recent study using mFISH showed a significant increase in the yields of translocations and complex chromosomal aberrations in Mayak workers with mixed external/internal exposures (Sotnik et al. 2014).

#### Exposure to ^232^Th

Thorium-232 (^*232*^*Th*) is an alpha emitter with a half-life of 1.45·10^10^ years. It is a primordial radionuclide and the parent of a decay chain which contains 10 alpha- and beta-emitters and ends with ^208^Pb. Two scenarios are considered here: exposure of Thorotrast patients and occupational exposure of workers in the NORM industry.

Internal exposure of ^*232*^*Th* is determined by in-vitro bioassay of urine samples, which may be complemented by analysis of faeces. Excretion rates of natural thorium should be evaluated for the population in the region of residence of the workers, to establish a background of non-occupational exposure. Thorium-232 cannot be detected directly by in-vivo measurement. Assessment of ^*232*^*Th* in lungs by measurement of the gamma emissions of progeny nuclides is not straightforward. It depends on equilibrium conditions in the source material to which the worker is exposed and on the biokinetic of the chain members in the lung (ICRP 2018).

##### Exposure of Thorotrast patients

Thorotrast is a suspension containing particles of thorium dioxide, ThO_2_, that was used as a radiocontrast agent in medical radiography from its introduction in 1931 until the 1950s. Kaul (1969) estimated that Thorotrast was used for diagnostic purposes in about one million cases around the world. Twenty-six publications, selected from more than 100 papers, were used for this study. The use of Thorotrast was reported in Germany (van Kaick et al. 1986), Sweden and Denmark (Faber 1973), Portugal (da Silva et al. 1986), Scotland (Buckton and Langlands 1973), England and Austria (Fischer et al. 1966; Dudley 1978), USA (Janower 1973) and Japan (Mori et al. 1986).

Thorotrast dosimetry is a problem of great complexity, partly due to the colloidal nature of ThO_2_ and partly due to the large number of different progenies of ^232^Th, the biokinetics of which differs from the biokinetics of the parent radionuclide. As ^232^Th and ^228^Th are alpha emitters, their progenies are able to escape from ThO_2_ aggregates, due to recoil and thus are translocated to other organs and also excreted from the body.

The distribution and retention of ^232^Th and its progeny were assessed by means of in vivo measurements (whole-body counting and scanning of the human body with collimated detectors), by measurement of activity of ^220^Rn in exhaled air from which ^224^Ra equivalent activity is calculated, by measurement of activity of ^212^Pb, ^212^Bi in blood and ^228^Ra, ^224^ Ra and ^212^Bi in excreta (Grillmaier 1964) and from autopsy samples. According to Kaul (1973), about 95% of intravascularly injected colloidal Thorotrast is retained by the organs of the reticuloendothelial system (RES) (59% liver, 29.5% spleen, 9.3% bone marrow). Only 0.7 and 0.1% are retained in the lungs and the kidneys respectively. The fractional retention of ^232^Th in the marrow-free skeleton is 4% on average. Ishikawa et al. (1999), revised data from 27 publications and added 140 Japanese cases; they estimated the relative partition of Thorotrast as 53% in liver, 14% in spleen, 24% in RBM and 8% in other organs. The hematopoietic system is thus exposed to alpha, beta and gamma radiation during the whole life of a patient. Alpha radiation is the dominant component of exposure. No external exposure is present.

Many authors estimated doses to individual organ and tissues of Thorotrast patients and, generally, they were in reasonable agreement (Hursh et al. 1957; Rundo 1960; Kaul 1964, 1965, 1973; Dudley 1967; Parr et al. 1969; Goldin et al. 1972; Travis et al. 1992,2).

The most comprehensive results on dosimetry of Thorotrast are in publications of Kaul (1965, 1973) and Kaul and Noffz (1978). Kaul (1965) described the procedure and assumptions under which doses are calculated. Table 2 is taken from Kaul and Noffz (1978) with an overview of dose rates in tissues of the reticuloendothelial system and in the kidneys. Together with the original values in rad year^−1^, the values are expressed in the current units of Gy year^−1^.

**Table 2:** Mean alpha dose rates in tissues of the RES and in the kidney of patients with long-term burdens of intravascularly injected Thorotrast (Kaul and Noffz 1978)

Organ	Injected amount (mL)	*F*^a^	Mean tissue dose rate	
			(rad year^−1^)	(Gy year^−1^)
Liver	10	0.852	12.5	0.125
	30	0.648	28.4	0.284
	50	0.522	38.1	0.381
	100	0.379	55.4	0.554
Spleen	10	0.498	41.5	0.415
	30	0.321	80.3	0.803
	50	0.285	118.8	1.188
	100	0.227	189.2	1.892
Red bone marrow	10	0.972	3.8	0.038
	30	0.919	10.7	0.107
	50	0.871	16.8	0.168
	100	0.766	29.6	0.296
Kidneys	10	1	0.17	0.0017

^a^Fraction of emitted α-energy escaping the aggregates

The values in Table 2 refer to a steady-state conditions, are valid for pure intravascular injection of Thorotrast and cannot be applied to cases with important perivascular deposits. The dose are not linearly correlated to the injected amount, because colloidal ThO_2_ deposits within the organs of the RES in aggregates or granules, giving rise to substantial self-absorption of α-particles. The mean fraction of emitted α-energy escaping the aggregates (*F*) decreases with increasing injected amount, and so do the dose rates, as shown in Table 2.

Very few papers contained chromosome analysis, usually in relation to applied amount of Thorotrast.

Fischer et al. (1966) reported data on whole-body counting (activity of ^212^Bi in the region of the upper abdomen, including liver and spleen) and chromosome analysis for 20 patients injected with Thorotrast during the years 1939 to 1947 and measured 19 to 27 years later. A significant correlation was found between the number of chromosome breaks and a value calculated by multiplying the activity of ^212^Bi measured in the upper abdomen region of the patients by the years since injection. This quantity was considered by the authors to be an indicator of the cumulated dose. In a paper by Buckton et al. (1967), information on amount injected, duration of exposure and results of chromosome analysis were reported for 36 Thorotrast patients. Another 25 cases were described in a study where dicentrics were measured 21–43 years post-injection (Teixeira-Pinto et al. 1979). Three further Thorotrast patients from the German Cancer Research Center were studied about 45 years after a single injection of Thorotrast (Tanaka et al. 1996) while Ishihara et al. (1978) followed for 9 years a patient exposed 26 years earlier with a known amount of injected Thorotrast.

In this analysis the results of the cytogenetic analysis were compared to the dose rate to the red bone marrow (RBM). Only Tanaka et al. (1996) presented direct doses to RBM. For the other papers, where the values of the injected activities are given (Buckton et al. 1967; Teixeira-Pinto et al. 1979; Ishihara et al. 1978), the data presented in Table 2 were used to evaluate the dose to the RBM. Alternatively for the patients reported by Fischer et al. (1966), injected activities were estimated based on the relationship between in vivo distribution of progenies of ^232^Th-^228^Ac and ^208^Tl and the injected amounts given in the papers of Kaul (1965) and Malátová and Dvořák (1971).

**Fig. 4:**
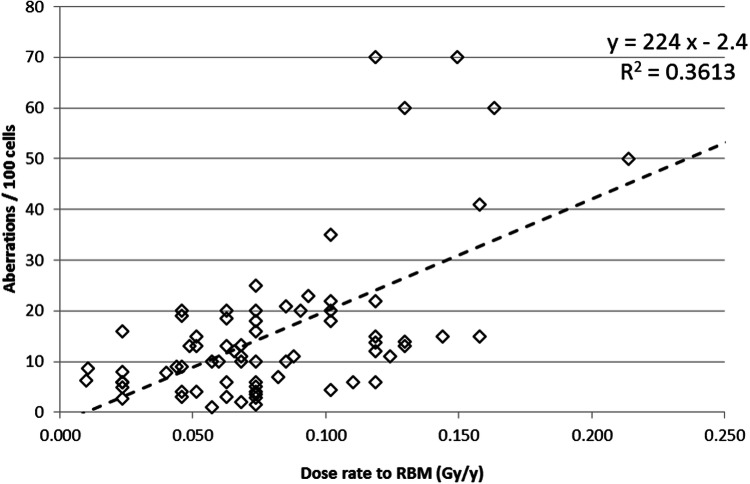
Summarized data of chromosomal aberrations compared with dose rate to RBM—males and females combined

Not all 84 available cases were considered in the analysis: cases were excluded when the amount of injected Thorotrast was not exactly stated or Thorotrast was used for retrograde pyelography or for perinasal sinus visualization and therefore, very low activity was present in the body; thus from 84 cases, only 72 cases were useful for further analysis. The relationship between chromosomal aberration frequencies and dose rate to the RBM is shown in Fig. 4. The correlation coefficient of *R* = 0.60 (*R*^2^ = 0.36) indicates a positive correlation between dose rate and effect. It should be noticed that actually correlation is expected between dose and effect; to assess such correlation the duration of exposure should be known.

There are large uncertainties associated with several parameters used to derive RBM doses. These include the content of ^232^Th in the injected colloid and the ratio of ^228^Th to ^232^Th activity. Also the variable distributions of Thorotrast among organs and tissues could differ from the original estimates (Ishikawa et al. 1999). Most publications do not consider these points.

A further complicating factor is that the cytogenetic endpoints used in these studies, mostly DCs and acentric fragments in T-lymphocytes, are not stable: such lesions would typically prevent successful cell division and would therefore not have been passed on from haematopoietic stem cells in RBM to the pool of long-lived lymphocytes circulating in the lymphatic tissues. Therefore, the damage reported in those studies that used peripheral blood, may largely reflect exposure of lymphocytes in the lymphatic system including the lymph nodes which were an important depot for Thorotrast, as mentioned earlier. Only one study (Tanaka et al. 1996) reports stable translocations data with a high level of translocations (from 3 to 8% of simple translocations).

##### Occupational exposure to ^232^Th

Workers are occupationally exposed to thorium in the industry handling naturally occurring radioactive materials (NORM). Brazil has one of the largest deposits of thorium in the world; thorium is usually produced from monazite sand containing 1–6% of ^232^Th (Juliao et al. 1994a). More than 13,000 workers are exposed in Brazil in the monazite cycle (monazite sand extraction plants, thorium concentration plants and gas mantle factories) and in conventional mining (Juliao et al. 1994b). Chronic occupational intakes occur in those facilities mainly via inhalation but also through ingestion (Juliao et al. 1994a; Dantas et al. 1994).

A risk of thorium incorporation also arises from inhalation of dust during the handling of the materials in the thorium industry using mineral Zircon sands (containing ^232^Th) e.g. in foundries. Intakes of ^232^Th could also happen by inhalation of aerosols produced by phosphate ores and fertilizers (Dantas et al. 1994).

Evaluation of committed doses (mSv) due to the intake of thorium is a difficult matter, and requires a correct interpretation of monitoring data. Individual monitoring of the thorium workers of this scenario study was carried out mainly by determination of the activity of thorium in faeces (also in urine samples) by alpha spectrometry. An alternative method would be using ICP-MS for determination of thorium concentration in urine. In vivo monitoring (gamma spectrometry) permits the determination of the activity of thorium daughters such as ^228^Ac (908 keV) and ^208^Tl (2614 keV) in the body (total body, lungs and head results were reported), but equilibrium in the ^232^Th chain must be confirmed. Finally, exhalation measurements of progenies of ^232^Th and air sampling monitoring are also available methods for intake estimation (Juliao et al. 1994b).

Regarding biokinetics, a thorium model is described in ICRP publication 69 (ICRP 1995). Inhaled particle size influences the metabolic behaviour of thorium isotopes inside the body. Typical AMADs reported are around 1 µm for the thorium industry, and chemical compounds present in thorium industrial facilities are thorium oxide (absorption type S) and thorium nitrate (absorption type M).

Regarding biological dosimetry, cytogenetic studies were carried out mainly for the analysis of the frequency of dicentric and ring chromosomes in lymphocytes from monazite workers in Brazil and the United States.

There are no publications where biological dosimetry doses and internal doses of the subjects are compared. Biological dosimetry studies of chromosome aberrations and individual monitoring results of body burden were reported, but without dose evaluations.

Results used for this study were reported in six publications (Costa-Ribeiro et al. 1975; Juliao et al. 1994a, 1994b; Lipsztein et al. 1989, 1992; Hoegerman and Cumming 1983).

A study of 67 thorium workers in Brazil from a monazite sand processing mill was published in 1975 (Costa-Ribeiro et al. 1975). Radiation hazards were assessed by sampling and measurement of airborne radioactivity, ^210^Po bioassay in urine and chromosomal analysis in peripheral blood lymphocytes. An increase in the level of the chromosome breakage was found in subjects who worked in the highly contaminated area of the plant and were more highly exposed to radiation from ^220^Rn and its daughters. But only a few workers had a slight increase in their urinary concentrations of ^210^Po, although results did not show a statistically significant difference between these values and those found in control population (Costa-Ribeiro et al. 1975).

The analysis of the frequency of chromosome aberrations (dicentric + centric ring chromosomes) in lymphocytes was reported for 47 American thorium workers and 3 external controls (Hoegerman and Cummins 1983). Three groups were established based on their emanating ^224^Ra and ^212^Bi body burdens. An increased frequency of dicentrics and ring chromosomes was found in US thorium workers with relatively high body burdens when compared with low burden cases and historical controls. The adjusted aberration frequency in the Brazilian study (Costa-Ribeiro et al. 1975) was higher than the frequency found in American workers. There was no positive correlation between duration of employment and aberration frequencies. Authors concluded that it would be possible that chromosome aberrations in US workers were induced by radionuclides inside their body and not by radiation resulting either from external sources or from inhalation of ^220^Rn or its daughters as may well have been the case in Brazilian population. In broad outline, the observed aberration frequencies in US workers are compatible with the results of the Brazilian study of Thorium workers (Costa-Ribeiro et al. 1975).

Another Brazilian study examined a plant where around 150 tons of monazite sand were processed per month, containing 1–6% thorium oxides (Lipsztein et al. 1989). A total of 146 workers were in the potentially exposed group. Regarding bioassay monitoring, the most sensitive method for detecting thorium exposure routinely is the analysis of faecal samples. Measurements were carried out after a closure of the facility or in a post-vacational period. In vivo monitoring results have to rely on assumptions about the behaviour of thorium progeny in air and in the body regarding equilibrium considerations of the thorium chain which are not always adequate. These analyses showed intakes above the annual limit of intake (ALI) recommended by ICRP Publication 30 (ICRP 1979). Besides internal exposures the workers were also exposed to external radiation.

A group of 72 individuals (presumably from the same facility) was used for a cytogenetic study, 51 having been exposed to the monazite with 39 of them from the “hot section” of the facility with risk of exposure to radiation at the workplace. A study of the frequencies of dicentrics and centric rings was made. There was a significant difference between the monazite group and the control group, but no difference was found between the “hot” and the “cold” sectors. There was no correlation between the frequency of chromosomal aberration and years of employment. It was concluded that abnormal frequencies of chromosomal aberrations in these Brazilian workers are linked to occasional external acute radiation exposure rather than to chronic internal exposure.

Another publication (Lipsztein et al. 1992) presented the average annual external dose for workers of the hot-section of a monazite treatment plant in Brazil as 14 mSv in the period 1973–1984, comparing with the 2 mSv for individuals at the cold-section. All workers received annual doses < 50 mSv (annual limit for effective dose at that time). Monitoring for internal exposure was not performed on regular basis, but measurements of the activity in faecal and urine samples from a representative group of workers indicated chronic exposure and some intakes above the investigation levels. Cytogenetic studies were also conducted at this plant. Results showed a statistically significant difference in the proportion of dicentrics, centric rings, aneuploidy cells and chromosomal breaks between the control group and the hot-section workers. The presence of chromosomal aberrations in this group cannot be explained by the external dose. Cytogenetic results may indicate a significant dose due to internal exposure (Lipsztein et al. 1992).

Regarding the biological dosimetry approach, the conclusion is that cytogenetic evaluations of industrial thorium exposure have not been studied very carefully. Cytogenetic studies in Brazil (Lipsztein et al. 1989) and US (Hoegerman and Cumming 1983), showed no positive correlation between chromosome aberration frequencies and duration of employment.

#### Therapeutic use of ^131^I

Inorganic iodide accumulates in the adult human thyroid, where it is used for the synthesis of the thyroid hormones thyroxine (T4) and triiodothyronine (T3), involved in the regulation of metabolic processes. Extrathyroidal iodine is distributed rapidly throughout the extracellular fluid and is rapidly excreted, mainly via the renal pathway. The isotope ^131^I is used mainly for therapeutic purposes in nuclear medicine. It has a half-life of 8.02 days and emits both gamma and beta radiation. The most abundant gamma emission is at 0.364 MeV (emission probability: 81.2%), and the most abundant beta emission has a maximum energy of 0.606 MeV (emission probability: 89.4%).

A total of 67 manuscripts were reviewed. The analysis here was mainly concentrated on the therapy of patients with differentiated thyroid carcinoma (DTC), in particular patients receiving radioiodine treatments for the ablation of thyroid remnant tissues after a nearly total thyroidectomy. In these patients uptake of iodine in the thyroid is very low, and a homogeneous whole-body irradiation can be assumed.

The Dosimetry Committee of the European Association of Nuclear Medicine EANM has developed a standard operating procedure (SOP) for the evaluation of absorbed dose and dose rate to the blood for DTC patients (Lassmann et al. 2008). The individual activity curves in whole body and blood are determined for each patient by means of measurements of the external dose rate and of the activity concentration in blood samples sequentially withdrawn from the patient, respectively. From these curves it is possible to estimate the dose rate and the committed dose to the blood over a given period, and also to differentiate between the separate contributions of beta and gamma emissions to the total dose.

Only a few studies (Lassmann et al. 2010; Eberlein et al. 2016) have used this SOP to evaluate individual blood doses to the patients and to compare them with a biological assay, specifically, the formation of γ-H2AX and 53BP1^3^ radiation induced foci (RIF). In vitro studies with blood samples to which a ^131^I-solution was added (Eberlein et al. 2015) have shown a linear relationship between the absorbed dose to blood due to internal irradiation (calculated using Monte Carlo techniques) and number of RIF per cell (for a dose range up to 95 mGy). However, the in vivo DNA damage repair mechanism has been found to be very rapid, with 76% of the damage being repaired with a characteristic half-life of 2.1 h and the rest with a half-life of about 17 h (Eberlein et al. 2016), so that this assay can hardly be effectively used in a retrospective dosimetric study, even in the immediate aftermath of an accident.

In several studies different biological and EPR dosimetry assays were used. However, in those studies a thorough physical dosimetry/individual assessment of the blood dose as recommended by EANM was not performed, thus invalidating the analysis.

Some studies just limit themselves to report the increase before and after therapy (Doai et al. 2013). Cancer patients are indeed known to have often higher aberration frequencies than healthier control persons (Iarmarcovai et al. 2008). Therefore, studies are generally conducted taking blood samples of radioiodine patients before and after therapy. A significant increase in the signal (DCs, MN, translocations) after the therapeutic treatment is reported in the majority of cases; however, authors often compare this increase with the administered activity of radioiodine and not with an individual (physical) estimation of the internal radiation dose. This might be misleading, as a standard activity of 3.7 GBq is generally administered to the patients, but it is known that the individual dose of the patients indeed might vary over a large range even if the same activity was administered. Hänscheid et al. (2006) report a variation of more than a factor of five for the calculated absorbed blood doses per unit of administered activity. Very important variables in this respect are indeed the patient size (mass), the amount of remaining thyroid remnant tissue left after surgery, the retention time in the remnant tissue and the renal clearance. In other cases, a patient-specific dose (which might be, according to the case, the dose to the thyroid, to the blood or the whole-body/effective dose) was calculated based not on the individual characteristics of the patients, but using reference values recommended by ICRP or by the Committee on Medical Internal Radiation Dose (MIRD) of the Society of Nuclear Medicine and Medical Imaging.

It is therefore not surprising that many of these studies do not find a correlation between the biological endpoints observed individually in each of the patients and the administered activity or the dose calculated with standard assumptions. In addition to the individual variations due to the biokinetic and anatomical characteristics of the patients, a further factor of uncertainty may be the low number of cells analyzed for the biological assay.

Similar considerations are valid when other therapies are considered, e.g. treatment of hyperthyroidism or therapy of the autonomous thyroid nodule. In patients treated for this disease the uptake in the thyroid is indeed not negligible and may vary significantly between subjects, as well as the retention time. In these cases the assumption of uniform whole-body exposure is therefore no longer valid.

Unfortunately the published data are not sufficient or not appropriate for a correct individual comparison of the physical and biological dosimetry estimates in thyroid cancer patients. Moreover, the comparison is made even more difficult by the fact that different calibration protocols have been applied, mainly developed for external gamma exposures and using the most disparate beam qualities. It can anyway be observed that the range of the published estimates of blood doses after administration of 3.7 GBq ^131^I (0.15–0.85 Gy), based on standard dose coefficients from ICRP and on individual dose studies, overlaps well with the range of 0.27–0.73 Gy estimated by means of cytogenetic assays such as DC, MN or FISH translocations (ICRP 1988, 2015a; Lassmann et al. 2010; M'Kacher et al. 1996, 1997, 1998; Nascimento et al. 2010; Puerto et al. 2000; Serna et al. 2008; Violot et al. 2005; Watanabe et al. 1998). Deluca (2005), Llina Fuentes (2006) and Vallerga (2008) showed a reasonable agreement between red marrow doses and cytogenetic doses (dicentrics and translocations) in patients with thyroid cancer.

Other studies show that in the case of therapies with repeated treatments with ^131^I, resulting in successive delivery of high blood doses, like for example in patients with metastases, the increase of chromosomal aberrations is not simply additive (M'Kacher et al. 1998). In addition to individual variation of iodine retention, the renewal of lymphocytes and different fading of the aberrations introduce large potential sources of error and make the dose assessment of thyroid cancer patients after several therapies very complex.

Finally, studies were conducted also in patients suffering with hepatocellular carcinoma and treated with ^131^I-Lipiodol. Monsieurs et al. (2003) were not able to find a significant correlation between the equivalent total body dose ETBD^4^ estimated by micronuclei assay and any of organ doses calculated according to the MIRD formalism or directly from the planar total-body scans using MonteCarlo methods. According to the authors, one possible explanation of that is the hypersplenism observed in these patients. As a consequence of this overactivity of the spleen, blood cells are removed too early from circulation, so that biological dosimetry results based on the MN assay are not reliable in these patients. On the contrary, De Ruyck et al. (2004) found very good agreement between the physical estimates of the dose to the blood and the results of biological dosimetry with dicentrics.

### Incorporation of mixtures of radionuclides

#### Semipalatinsk scenario: intake of ^239,240^Pu, ^137^Cs and ^89,90^Sr

In addition to plutonium and caesium, which were already presented before, in this scenario we have also strontium, a radionuclide with three isotopes of interest for internal dose assessments: ^85^Sr (gamma emitter), ^89^Sr and ^90^Sr (beta emitters). Analysis of ^90^Sr is usually carried out by LSC (liquid scintillation counting). The physiologically-based recycling model for strontium is presented in ICRP Publication 67 (ICRP 1993). Trabecular and cortical bone are the main sites of deposition and retention of strontium after intake and absorption to blood.

The USSR Semipalatinsk Nuclear Test Site (STS), is located in the north-eastern part of the Republic of Kazakhstan and covers an area of about 19,000 km^2^. The USSR performed 498 nuclear tests at the STS, including 26 surface and 92 atmospheric nuclear explosions carried out between 1949 and 1962 (Gusev et al. 1997). After, the signing of the Limited Test Ban Treaty in 1963, the tests at STS were restricted to underground detonations with little or no environmental contamination outside the STS. The last nuclear explosion at the STS took place in 1989.

The total energy released from all explosions during the 40-year-test period was 17.4 Mt of TNT equivalent (Tanaka et al. 2000). The atmospheric explosions contributed to this number with 6.6 Mt of TNT equivalent (UNSCEAR 2000). The explosions conducted on the surface and in the atmosphere greatly influenced the radiation exposure conditions mainly due to ^90^Sr, ^137^Cs and ^239,240^Pu for several hundred thousand people. Five atmospheric tests of September 1949, September 1951, August 1953 and March and August 1956 contributed to more than 85% of the doses to the population near the nuclear site (Gusev et al. 1997; Grosche 2002), including both external and internal components as estimated from environmental measurements and from calculations made on the basis on fallout trajectories. However, there was a considerable heterogeneity in exposures even in the same settlements, and thus uncertainty in the individual exposures. Moreover, after the dissolution of USSR, the control of the entrance to STS was no longer under military control, and there are indications that members of local populations entered the area. The population exposure was estimated to derive from two components; external irradiation during the explosions and the external gamma doses from the fallout. Based on environmental dosimetry, OSL in the bricks and EPR tooth enamel studies it was concluded that approximately 90% of lifetime doses to the population was due to external exposures during the first years after explosions (Gordeev et al. 2001). This evaluation, however, does not take into consideration internal contamination with short life radionuclides, especially iodine isotopes (Simon et al. 2003). Approximately 10% of the total lifetime doses have been attributed to doses from internal exposure primary due to ingestion of radionuclides, in later years after explosions (Stepanenko et al. 2006; Semioshkina and Voigt 2006). Seven artificial radionuclides are detected in the area around STS (^241^Am, ^57^Co, ^137^Cs, ^95^Zr, ^95^Nb, ^58^Co, and ^60^Co). Recently the external dose estimate to the population around the STS from these radionuclides was estimated to be under the annual limit of 1 mSv year^−1^ (Taira et al. 2013).

All the estimations of doses described above had been derived from the average group doses for the most affected settlements (Gordeev et al. 2002). In the first four decades of nuclear testing direct individual measurements of the radiation burden to the members of the overexposed population were not carried out either by bioassay or external retrospective dosimetry assays. The studies by biological methods (Ilyinskikh et al. 1997, 1998; Tanaka et al. 2000, 2006; Testa et al. 2001; Stephan et al. 2001; Salomaa et al. 2002; Chaizhunusova et al. 2006; Takeichi et al. 2006) or EPR or TL retrospective dosimetry (Ivannikov et al. 2006; Stepanenko et al. 2006, 2007; Zhumadilov et al. 2006, 2013; Sholom et al. 2007) were not made until the 1990s. Published reports in English language literature are available only after 1995.

Few reports are available on individuals' internal contamination measurements, but unfortunately, no external dosimetry data are available for these people.

The first WBC study on the internal contamination related to 765 individuals living in two villages, Mostik and Maisk did not detect contamination with ^137^Cs. The individual committed effective dose E(50) due to exposure to ^137^Cs was less than 12 µSv, which is the value of E(50) related to the detection limit for adults of the WBC installation (300 Bq) employed in the survey. However, the bioassay in vitro of 105 individuals showed that the urinary excretion rate of ^90^Sr was significantly higher than the excretion rate of a control group. The estimated maximum exposure from ^90^Sr in 1995 was about 5 μSv assuming continuous ingestion; the Sr excretion of Maisk population was mainly due to uptake from foodstuff in 1995. No detectable incorporation of other radionuclides (^239,240^Pu, ^234,238^U) have been observed in the 105 subjects from these two villages (Hille et al. 1998).

Higher internal doses were calculated in another study based on whole-body counting. The estimated maximum internal doses were in the range of 13–50 µSv year^−1^ for ^137^Cs and in the range of 30–500 µSv year^−1^ for ^90^Sr (Semiochkina et al. 2004; Semioshkina and Voigt 2006).

A direct evaluation of internal dose was performed by the α-ray spectrometry determination of ^239,240^Pu and uranium in human bones samples from residents in areas near the STS. From the measured concentration of radionuclides, the initial estimated total intake and the committed effective dose estimates were 10 Bq and 0.2 mSv for ^239,240^Pu, while the annual intake of total ^234,235,238^U and the committed effective dose were calculated to be 30 Bq and 0.1 mSv (Yamamoto et al. 2006).

Several biological dosimetry studies with the conventional cytogenetic techniques (by unstable chromosome aberrations: DC and MN assays) were performed about five decades after the beginning of the nuclear tests in 1949. These studies suggested that the current increased levels of unstable-type chromosome aberrations in the inhabitants of the most contaminated areas were mainly due to internal exposure (Tanaka et al. 2000, 2006; Testa et al. 2001; Takeichi et al. 2006) but these methods were not appropriate for long-term individual retrospective dose assessment.

In reviewing studies on stable chromosome aberrations by FISH, the yields of translocations reported in two different papers for individuals living in villages close to the STS did not differ from controls (Stephan et al. 2001; Salomaa et al. 2002). By contrast, another study reported elevated frequencies of stable translocations for the Dolon population, identified as the most highly overexposed subjects (Simon et al. 2003), corresponding to a dose of 0.18 Sv (Chaizhunusova et al. 2006).

Concerning EPR measurements, Zhumadilov et al. (2013) reviewed the literature results on individual absorbed doses in people living in 14 settlements in the Semipalatinsk region. The methods used for these EPR measurements of dose had been validated through an international exercise (Hoshi et al. 2007; Ivannikov et al. 2007). However, the protocols were not harmonized or at least this cannot be found in the open literature. The average excess doses were 141 ± 37 mGy for Dolon residents whose tooth enamel was formed before 1949 and completely formed by 1962 (when atmospheric testing was stopped) and 25 ± 11 mGy for younger residents. For the population of the most studied village of Dolon, the EPR dosimetry results were close to the doses obtained by FISH. This correlation was also highlighted in the 3rdWorkshop on the Semipalatinsk Nuclear Test Site (Stepanenko et al. 2006). However, it has to be stressed that there are very limited data on individual internal dose monitoring by bioassay for Dolon residents. It should be noted here that direct comparison of the FISH and EPR doses is not possible, due to the high degree of uncertainties around what these assays are measuring, and indeed whether the most appropriate calibration curves have always been applied, in this complex exposure scenario. Both EPR and FISH measurements will likely represent a combination of internal (long term) and external doses, however, the relationship between total delivered dose and dose measured by these techniques is not always clear (Ainsbury et al. 2014; Fattibene and Callens 2010).

In particular, referring to EPR, there is a knowledge-gap because no analysis has been made (at least reported in the open literature) about a possible correlation between the concentration of the bone-seeking radionuclides (e.g. ^239,240^Pu, U ad ^90^Sr) in tooth enamel and dentine and the accumulated dose measured by EPR in teeth.

Overall, the dose estimates based on the internal contamination measurements and biological and EPR dosimetry are generally lower than those obtained by historical reconstruction of external exposure rate (range of 1–4.5 Gy) (Gusev et al. 1997; Gordeev et al. 2002).

#### Techa river scenario: intake of ^89,90^Sr and ^137^Cs

As a result of the activities of the first Soviet plutonium production radiochemical plant operated by the Mayak Production Association, large territories of the Southern Urals were exposed to radioactive contamination. From 1949 to 1956 about 30,000 people living on the banks of the Techa river, in the Southern Urals region, were exposed due to waterborne radioactive releases of short- and long-lived fission products into the Techa river. Primary exposure resulted from external irradiation from ^95^Zr, ^95^Nb, ^103^Ru, ^106^Ru and ^137^Cs deposited in the sediments and floodplain soils along the shoreline of the Techa river and internal dose from the ingestion of ^89,90^Sr and ^137^Cs (Degteva et al. 2012; Shagina et al. 2012).

Residents of the riverside communities located far downstream of the site of release were mostly internally exposed to ^89,90^Sr from drinking water and contaminated foodstuffs while the external γ-ray exposure was significant in the upper reaches of the Techa region where the most ^137^Cs from releases was absorbed by sediments and soils (Shagina et al. 2012). The Techa River Dosimetry System (TRDS) has been developed to provide estimates of individual dose received by the members of the Techa River Cohort (Degteva et al. 2019).

The estimated doses from ^90^Sr are supported strongly by about 23,500 measurements made with a tooth β-particle counter, 1200 measurements of bones collected at autopsy, and about 30,000 measurements made with a special WBC that detects the bremsstrahlung from ^90^Y. In the TRDS, the mean doses to the RBM were reported to be 0.35 Gy (in the range up to 7.9 Gy), while the mean stomach dose was 0.059 Gy (range up to 1.13 Gy) and the mean muscle dose was 0.052 Gy (range up to 1.0 Gy) (Degteva et al. 2019). Thus, the doses to the majority of extra-skeletal tissues for the Techa region residents were comparable and significantly lower than RBM doses.

**Fig. 5:**
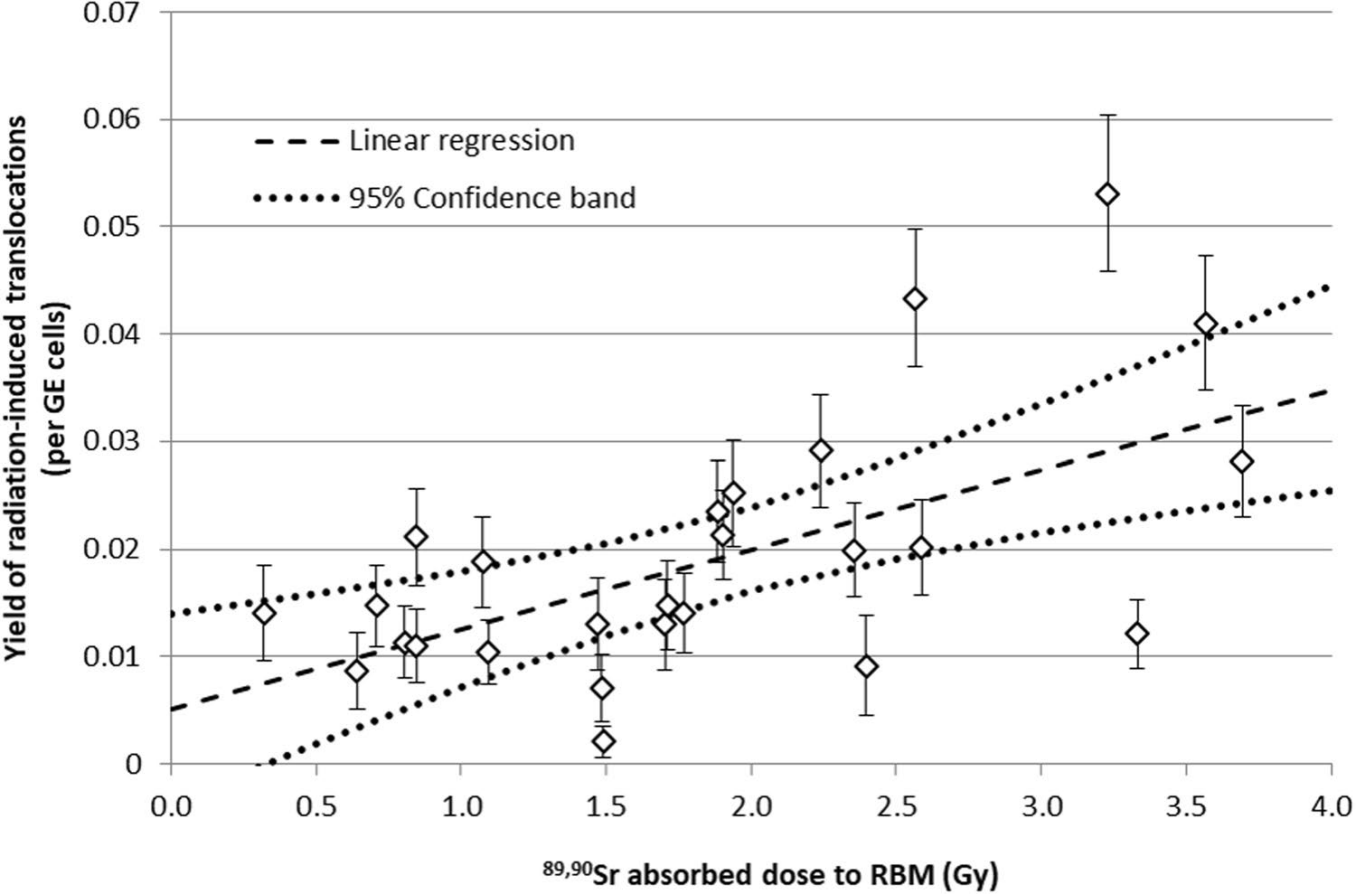
Linear dependence of radiation-induced translocation yields measured for 26 donors on their individual RBM dose from strontium radioisotopes (Vozilova et al. 2014). Bars indicate SE. Dashed lines indicate 95% confidence intervals of the linear regression

Vozilova et al. (2012, 2014) reported a FISH study on Techa residents; the group of donors comprised 26 persons who were predominantly exposed to ingested ^89,90^Sr (contribution to the total RBM dose exceeded 95%). The FISH assay was supported by individual measurements of ^90^Sr-body burdens made with the WBC. As shown in Fig. 5, a significant linear dependence (*R* = 0.61, *p* < 0.001) between the radiation-induced translocations frequency and individual ^89,90^Sr doses to the RBM derived from the WBC measurements was reported (Vozilova et al. 2014). The range of individual cumulated RBM absorbed dose was from 0.3 to 3.7 Gy. The linear dose relationship of 0.007 ± 0.002 translocation GEcell^−1^ Gy^−1^ was assumed to be the strontium-specific dose–response coefficient for the RBM exposure. This value is approximately half of the dose–response coefficient 0.015 translocation GEcell^−1^ Gy^−1^ obtained in vitro from external gamma exposure and recommended for retrospective biodosimetry (Edwards et al. 2005).

The second group in the study included 71 persons with mixed external exposure and internal exposures due to ^89,90^Sr and ^137^Cs (Degteva et al. 2015). Translocations from ^89,90^Sr were calculated from the individual RBM dose (derived from the WBC measurements) and use of the ^89,90^Sr-specific dose–response coefficient derived as described above (Vozilova et al. 2014). The dose from external exposure and ^137^Cs intake was assessed from the total number of translocations by subtracting the contributions from spontaneous translocations and translocations induced by ^89,90^Sr. The FISH-based dose estimates for RBM were derived with use of the dose–response coefficient recommended by Edwards et al. (2005) assuming that the translocations observed in peripheral blood lymphocytes of the studied subjects were induced by irradiation of progenitor cells in the bone marrow a long time ago (Vozilova et al. 2014). The values were reported to be comparable with corresponding TRDS-based dose in soft tissues from external exposure and ^137^Cs intake (Degteva et al. 2015).

A recent re-evaluation was reported of the combined results of FISH studies undertaken during 1994–2012 for 178 persons exposed on the Techa riverside (Tolstykh et al. 2017, 2019). Absorbed doses in RBM and soft tissues were estimated using an improved strontium biokinetic model (Shagina et al. 2015) and the latest version of the Techa River dosimetry system (Degteva et al. 2019). To interpret the FISH results, the authors suggested that some portion of T-cells represented the offspring of thymocytes which matured before the ^89,90^Sr intake. If the dose to the lymph nodes is much lower than to the RBM (as for ^89,90^Sr intake), the dose to circulating lymphocytes should also be less than the dose to RBM. A model of T-lymphocyte irradiation was proposed by the authors that addressed: (1) irradiation of the part of T-cell progenitors in RBM; (2) irradiation of T-lymphocytes in extra-skeletal lymphoid tissues, and (3) mature T-lymphocyte recycling in RBM (Tolstykh et al. 2017). According to the model estimates, the doses to T-lymphocytes calculated for the persons exposed to ingested ^89, 90^Sr, were about 0.4–0.8 of RBM dose depending on person’s age (Tolstykh et al. 2017). Statistical analysis of the combined FISH data from the Techa River showed that the use of doses to T-lymphocytes (instead of RBM dose) resulted in the dose–response coefficient of 0.0118 ± 0.0012 translocation GEcell^−1^ Gy^−1^ (Tolstykh et al. 2019), which agrees well with the value of 0.0116 ± 0.0016 estimated for radiation workers from the Sellafield nuclear facility under external exposure (Tawn et al. 2015).

EPR measurements of teeth from the Techa river population were carried out from 1993 to 2012, so that a protocol of a posteriori harmonization of measurements from different laboratories and different calendar periods had to be developed (Volchkova et al. 2011), including the subtraction of background levels evaluated from EPR measurements of teeth from donors who lived in uncontaminated areas of the Urals region.

EPR investigations of teeth from the Techa river population have identified significantly high absorbed doses in tooth enamel due to incorporation of ^90^Sr in the calcified dental tissues (Tolstykh et al. 2003; Shishkina et al. 2011). Because of the biokinetics in teeth, these doses are only absorbed in tooth enamel and are not associated with exposures to other organs or the whole body. Tooth samples from a number of residents of Upper Techa were analyzed using both thermoluminescent (TL) dosimetry (Göksu et al. 2002; Shishkina et al. 2014) and EPR techniques to assess ^90^Sr concentration and related doses and dose rates separately in dentine and enamel (Shishkina et al. 2016). These results were used for calculating internal doses in enamel and interpretation of the measured absorbed dose by EPR.

The typical mean values of external dose for the Upper Techa region vary from 510 to 550 mGy for villages located at stagnant reservoirs accumulating the radionuclides to 130–160 mGy for villages located at the banks of free-flowing river. The upper bound of individual estimates for both EPR and FISH methods is equal to 2.2–2.3 Gy. In the middle and low Techa area mean doses of external exposure were 30–80 mGy. Authors reported that the TRDS-based absorbed external doses in tooth enamel and muscle are in agreement with EPR and FISH-based estimates within uncertainty bounds (Degteva et al. 2015).

## Discussion and conclusions

Although this is clearly a complex topic, the following observations can be inferred from the results presented here for the selected case scenarios involving the application of retrospective dosimetry techniques (biological and EPR methods) after accidental internal exposures.

First, it is difficult to obtain clear and straightforward indications from the available evidence and to match data presented and published at different times, since biological and EPR dosimetry and internal dose estimates were often performed in different studies published independently. Sometimes, the same affected population groups have been studied in very different years or decades, producing data that cannot be correlated with each other. Internal contaminations indeed seldom occur alone, especially in emergency situations. In nearly all scenarios presented, incorporation of radionuclides was accompanied by external exposures, which were usually responsible for the greater portion of the received dose. Some evidence was found in some of the mixed scenarios studied here that internal exposures probably occurred, as retrospective doses did not justify external exposure only. However, it is not possible to infer from these studies specific conclusions valid for incorporated radionuclides alone, since for the moment no assay is available which can discriminate between radiation of different types and different LETs on the basis of the type of damage induced.

Ideally, assays and endpoints would be needed which could "fingerprint" the radiation type and thus provide separate information on the contribution of external and internal doses. The cytogenetic techniques have been developed and optimized generally for acute external exposures and calibration curves have been generated using external photons. However, doses from incorporated radionuclides are actually chiefly delivered by the emitted alpha- and beta-particles, although a gamma and X-ray photon component is nearly always present. In addition, internal contaminations are typically extremely non-uniform, due to the inhomogeneous distribution of the radionuclides in the different body tissues. The exposure is prolonged over potentially a long period of time, and the dose rate is not constant but varies over time depending on the physical half-time of the incorporated radionuclides and on their time-dependent distribution and retention in the different tissues. Only in very rare cases of accidental intakes of radionuclides (e.g. subjects exposed to tritiated water) have appropriate calibration curves been constructed (Lloyd et al. 1986; Prosser et al. 1983). The critical issue is how to interpret measurement results in the presence of mixed exposures, when the relative contribution of different radiation types and different exposure modalities (internal vs. external, acute vs. chronic) are not known.

Some publications have used animal models to study the cytogenetic damage induced after internal contaminations (Little and Lambert 2008; Roch-Lefèvre et al. 2016). The overall conclusion of these authors is that less damage is produced after internal exposure than after acute external exposure. When converting the yield of aberration to the dose using calibration curves established with external radiation, the corresponding doses will be consequently underestimated. In vitro studies are, therefore, still needed to characterize the response of the biological assays to a prolonged exposure to internalized particle emitters, among others how chronic irradiation affects the damage repair mechanisms and ultimately the linearity of the method. An important matter to take into consideration is the appropriate dose quantities when interpreting monitoring data for internal dosimetry evaluations (e.g. dose to the blood) to be compared with biological and EPR dosimetry results. How to calculate them is under discussion.

In some of the selected scenarios (tritium, Thorotrast, radioiodine), the incorporation of radionuclides was the only or the dominant source of exposures. The case of administration of radiopharmaceuticals in diagnostic and therapeutic nuclear medicine is not representative of an emergency situation (exposure conditions such as administered activity and biodistribution are well known, so that doses can be calculated using the reference procedures), however, the presented studies can be helpful in assessing the feasibility of the use of biological dosimetry in the assessment of internal dose. Unfortunately, a number of studies relied on the use of biological assays which are not optimized for the detection of ionising radiation exposure; therefore, they have been excluded from this review. Moreover, often correlations are sought between biological and EPR dosimetry results (which are estimated individually) and the incorporated activity or the retention in body or excretion measured at a specific time after intake. These quantities, however, can be related to the dose if knowledge of the individual biodistribution and anatomical characteristics of the subject is available. If however generic, reference dose coefficients or bioassay functions, not related to the specific study subjects, are used, it is not surprising that no correlation is found with the individually assessed EPR or biological dosimetry estimates.

However, considering those studies that were considered reliable both with regard to biological dosimetry and to internal dose estimation, it can be cautiously stated that the biological dosimetry assays and EPR techniques proved to be most applicable in cases when the radionuclides are almost homogeneously distributed in the body (e.g. tritium, caesium, iodine in patients with no thyroid gland). No compelling evidence was obtained in other cases of extremely inhomogeneous distribution (e.g. insoluble compounds of actinides that remain a long time, predominantly in the lungs).

In addition to all the limitations indicated so far, one must consider that in the case of internalization of radionuclides, and even more in mixed internal exposures, an adequate assessment of uncertainties can be hugely complex and requires a detailed formal consideration of the total uncertainty budget on a case by case basis. The key sources can be identified in the paucity of human data and in the potential inter-individual variation (Suzuki et al. 2002; Paquet et al. 2016). Nevertheless, formal or even informal assessments, where information is lacking, are essential to ensure the quality and validity of the dose estimation techniques applied (Ainsbury et al. 2014, 2018).

Finally, the conclusions presented here need to be weighed against the scant availability of proper studies on retrospective assays in cases of internal contamination only. However, this also means that in real cases, a mixture of both external and internal exposures will be encountered most of the times, and retrospective dosimetry needs to be optimized and developed in order to be able to deal with this situation. This is only possible if specific targeted research in this field is conducted and supported. Further developments may help to solve the problems identified during this survey and thus contribute to reduce the related uncertainties. This is needed before new studies (e.g. clinical studies) may be proposed as well as the existing cases are re-evaluated.

## Footnotes

1 See also all other contributions in the issue 93 of the International Journal of Radiation Biology, entirely dedicated to the RENEB network.

2 See also all other contributions in the issue 63 of Health Physics, entirely dedicated to the evaluation of a Thorotrast patient.

3 The technique based on 53PB1 RIF is not a recognized technique in biological dosimetry, and no standardization work has been yet conducted on it. For this reason no details on this technique are given in this review.

4 The equivalent total body dose is defined as the absorbed dose of ionising radiation, which, if received homogeneously by the whole body, would produce the same signal yield as observed in the patients.
